# Analysis of the benefits of imputation models over traditional QSAR models for toxicity prediction

**DOI:** 10.1186/s13321-022-00611-w

**Published:** 2022-06-07

**Authors:** Moritz Walter, Luke N. Allen, Antonio de la Vega de León, Samuel J. Webb, Valerie J. Gillet

**Affiliations:** 1 Information School, University of Sheffield, 211 Portobello, Sheffield, S1 4DP UK; 2 Lhasa Limited, Granary Wharf House, 2 Canal Wharf, Leeds, LS11 5PS UK

**Keywords:** QSAR, Imputation modeling, Multi-task modeling, Toxicity prediction, Model evaluation

## Abstract

Recently, imputation techniques have been adapted to predict activity values among sparse bioactivity matrices, showing improvements in predictive performance over traditional QSAR models. These models are able to use experimental activity values for auxiliary assays when predicting the activity of a test compound on a specific assay. In this study, we tested three different multi-task imputation techniques on three classification-based toxicity datasets: two of small scale (12 assays each) and one large scale with 417 assays. Moreover, we analyzed in detail the improvements shown by the imputation models. We found that test compounds that were dissimilar to training compounds, as well as test compounds with a large number of experimental values for other assays, showed the largest improvements. We also investigated the impact of sparsity on the improvements seen as well as the relatedness of the assays being considered. Our results show that even a small amount of additional information can provide imputation methods with a strong boost in predictive performance over traditional single task and multi-task predictive models.

**Supplementary Information:**

The online version contains supplementary material available at 10.1186/s13321-022-00611-w.

## Introduction

There has been a surge of interest in applications of multi-task prediction methods in the chemoinformatics field during the last few years. Their main advantage is the ability to predict multiple biological endpoints of interest using a single model. Early work on multi-task learning showed that training a model on several tasks (sometimes even unrelated tasks) simultaneously can lead to improved performance compared to single task learning [1]. Multi-task modelling gained attention in the chemoinformatics field in the context of deep learning for QSAR modelling. In both the Tox21 challenge and the Kaggle challenge organized by Merck, the winning approaches were based on multi-task deep neural networks (DNNs) [2, 3], highlighting the potential of these approaches. The inputs to the DNNs were chemical features and the outputs bioactivity profiles. In the work by Ma et al., a comparison was made of single task and multi-task DNNs on the Merck Kaggle dataset with the multi-task DNNs outperforming the single task DNNs, except for the two largest of the 15 assays. Similar findings were reported by Mayr et al. who compared single task and multi-task DNNs on the Tox21 dataset and found that the multi-task DNNs resulted in higher ROC-AUCs for nine of the 12 assays. A significant benefit of multi-task DNNs, over more traditional method such as random forests (RF), is their ability to be trained on sparse activity datasets. Sparse datasets are those where not all compounds have been tested on all targets, and they are a common occurrence in bioactivity databases. Macau is another technique recently introduced in QSAR that is also able to train on sparse datasets [4–6].

Imputation is a technique for filling gaps in a dataset based on the existing data and is widely used to substitute empty attribute or descriptor values. Perhaps the simplest imputation method is to use the mean value of the available data. More sophisticated techniques include the use of machine learning models to predict the missing values, where the models are trained using all the other attributes. Varnek et al. introduced imputation to fill missing bioactivity values in multi-task QSAR modelling in 2009 [7]. They compared the performance of single task artificial neural networks (ANNs) with multi-task ANNs and Feature Nets (FN). The multi-task ANNs used only chemical descriptors as inputs, whereas, in the FN models, the activities of compounds in related tasks were used as features in addition to the chemical descriptors. The FN involved two training steps. First, an ANN model was built for each task independently and used to predict the activity values for that task for the compounds of the test set. Second, the models were retrained using both chemical features and activity values, which were the predicted values generated in the first step. Thus, the predicted value of property B was used explicitly as an additional feature when predicting property A. The tasks considered by Varnek et al. were tissue-air partition coefficients for different tissues. They found that both multi-task ANNs and FN ANNs improved the performance over single task ANNs in most of the cases. In a more recent study, Sosnin et al. found FN to perform better than single task models, but slightly worse than multi-task ANNs when predicting acute toxicity [8]. In related work, Norinder et al. have used a method comparable to FN (i.e., using predicted bioactivity profiles as additional inputs to a QSAR model) in combination with conformal prediction for predicting several cytotoxicity and bioactivity datasets and they also found improved performance compared to single task models [9].

Two other imputation approaches have been described more recently for regression problems: pQSAR and Alchemite. pQSAR [10] is similar in concept to FN and consists of a two-step procedure. In the first step, a RF regression model is trained for each assay based on chemical fingerprints as features and the models are used to fill the gaps in the sparse dataset. In the second step, a partial least squares model is trained for each assay on the profile of the remaining assays as obtained in the first step. To reduce the number of features, only assays related to the target assay are included. The algorithm was tested on a proprietary Novartis dataset (11805 assays) and a public ChEMBL dataset (4276 assays). The pQSAR model clearly outperformed RF models on both datasets. For the Novartis dataset, pQSAR achieved a median R^2^ (across all the assays) of 0.53 compared to 0.05 achieved by RF.

Alchemite is based on DNNs and was developed in a collaboration between Optibrium and Intellegens [11, 12]. The neural network uses chemical descriptors and the bioactivities of the assays as inputs. Missing input data is initially replaced by the mean value for the respective assay and predictions made by the network are used to update the missing values iteratively. The cycle continues until no further improvement in the predictions is observed. On a kinase dataset, the Alchemite method clearly outperformed RF models, a collective matrix factorization method (similar to Macau) and a multi-task DNN, and achieved approximately the same performance as pQSAR 2.0 [13]. A notable feature of Alchemite is its capability to express confidence in single predictions. This is achieved by training the network with several random initializations and using the standard deviation across different runs of the model as a measure of uncertainty. The confidence in the predictions is correlated with the accuracy and the performance can be increased by keeping only the most confident predictions.

Both of these recent imputation methods clearly outperformed single task and standard multi-task models. The benefit in performance of the models seems to come from the relations between different assays and the models’ capability to leverage these patterns. However, it is unclear what characteristics of a dataset cause this behavior and hence under which circumstances imputation approaches will be particularly effective.

In this study, we focus on FN as an imputation method and compare its performance with Macau and DNNs on single task and multi-task classification problems applied to sparse toxicity datasets. The focus on classification is motivated by common practice in the toxicity field whereby QSAR models are often used to establish whether compounds are potentially reactive towards measurable toxicity endpoints (hazard identification) [14]. Following an initial comparison of the performance of the methods, we seek to determine the factors that contribute to good performance in the imputation methods by examining different subsets of test predictions to assess which improved the most. We also tested whether the contribution of individual assays to the performance of imputation models could be modelled using information theory metrics. Our results confirm that imputation methods provide benefits over single and multi-task methods and are a very promising approach in chemoinformatics. In addition, we found the benefits of imputation techniques to be particularly high for compounds that had been measured in many of the assays and for compounds that were chemically dissimilar to compounds in the training set, where conventional QSAR models frequently fail to make reliable predictions. Furthermore, we show that information theory metrics can be used to identify auxiliary assays that are particularly useful for imputing data for a given target assay.

## Methods

### Datasets

Three in vitro toxicity datasets were used: the ISSSTY (Istituto Superiore di Sanità Salmonella Typhimurium) database containing data for the Ames test for mutagenicity, generated by the Italian National Institute of Health; the Tox21 dataset, generated by the Tox21 consortium; and the ToxCast dataset, generated by the EPA (U.S. Environmental Protection Agency).

The ISSSTY database [15] contains data for six different bacteria strains, designed to detect different mechanisms of mutagenicity, such as substitutions of DNA bases or deletions and insertions leading to a frameshift in the triplet code of the DNA. The Ames test is a well-established in vitro test for mutagenicity used in regulatory contexts [16]. Each of the six strains was tested with and without the addition of S9 mix to mimic metabolism of higher organisms, which leads to a dataset of 12 different toxicity assays. The outcome for a compound in each of the assays is either ‘active’, ‘inactive’ or ‘equivocal’ [17], due to the fact that the database may contain repetitions of the same experiment. In the original dataset, a compound was considered ‘active’ if it was active in more than 60% of repeated experiments for a given strain and ‘inactive’ if it was active in less than 40% of the experiments. Otherwise, the result was considered ‘equivocal’. To obtain a binary dataset, ‘equivocal’ activity values were removed from the data table.

Within the Ames dataset, each assay has an associated assay pair based on the presence or absence of the S9 matrix. A toxic outcome with S9 and a non-toxic outcome without, indicates that it is a metabolite of the tested compound that is the cause of the toxicity. Positive values with and without S9 indicate that metabolism is not required for toxicity, however, metabolism may also result in the loss of toxicity indicated by a positive result without S9 and a negative result with S9.

Different Ames strains can identify different types and mechanisms of mutation, for example, TA98 identifies frameshift mutations whereas TA100 identifies base-pair substitutions [18]. Understanding which strains are positive may reveal insights into the toxicity mechanism, which is not gained from a prediction of the overall call alone. There is some overlap between the types of mutation different assays measure that may be leveraged under a multi-task or imputation modelling strategy.

The Tox21 dataset [19] consists of three separate datasets named ‘training’, ‘testing’ and ‘final evaluation’, as used in the Tox21 QSAR modelling challenge (which is a subset of the total Tox21 dataset). For this study, the three datasets were combined to a single dataset. The dataset contains binary data (active or inactive) for 12 toxicity assays. Seven of these measure the activation of various nuclear receptors related to toxic effects, while the remaining five measure the activation of cellular stress pathways. These are assays that are typically conducted in preclinical toxicity screening of chemicals [20]. The Tox21 dataset contains two sets of matched assays: NR-AR with NR-AR-LBD; and NR-ER with NR-ER-LBD. These matched pairs contain the full receptor (NR-AR and NR-ER) or only the ligand binding domain of the receptor (NR-AR-LBD and NR-ER-LBD).

Table 1 describes the number of molecules and the proportion of actives for each assay in the Ames and Tox21 dataset.

**Table 1:** The Ames and Tox21 datasets

Ames			Tox21		
Assay name	Number labels (proportion)	Proportion actives	Assay name	Number labels (proportion)	Proportion actives
TA100	4627 (0.75)	0.26	NR-AhR	6810 (0.84)	0.12
TA100_S9	4350 (0.71)	0.32	NR-AR	7460 (0.92)	0.03
TA102	880 (0.14)	0.17	NR-AR-LBD	6991 (0.86)	0.03
TA102_S9	763 (0.12)	0.21	NR-Aromatase	6009 (0.74)	0.05
TA1535	2489 (0.40)	0.11	NR-ER	6367 (0.79)	0.11
TA1535_S9	2347 (0.38)	0.12	NR-ER-LBD	7199 (0.89)	0.04
TA1537	2081 (0.34)	0.12	NR-PPAR-gamma	6752 (0.83)	0.03
TA1537_S9	1998 (0.32)	0.12	SR-ARE	6121 (0.76)	0.16
TA97	1049 (0.17)	0.14	SR-ATAD5	7326 (0.91)	0.04
TA97_S9	1010 (0.16)	0.17	SR-HSE	6794 (0.84)	0.05
TA98	4345 (0.70)	0.24	SR-MMP	6074 (0.75)	0.15
TA98_S9	4055 (0.66)	0.29	SR-p53	7049 (0.87)	0.06
overall	6168 (0.41)	0.21	overall	8090 (0.83)	0.07

Each row contains the total number of molecules with an experimental label for the assay as well the proportion of active labels for the given assay. The last row (‘overall’) reports the number of unique compounds across all the assays (after the data processing steps described in ‘Data processing’). The proportion of labels in the last row indicates the completeness of the data table across all assays. The proportion of actives in the last row reports the proportion of actives among all available labels.

The ToxCast dataset [21] was downloaded from the MoleculeNet platform where it is provided with binary labels [22]. The ToxCast dataset represents a large-scale in vitro toxicity dataset, containing 8615 compounds and 617 assays (reduced to 7787 compounds and 416 assay in this study after the standardization and filtering steps described below were applied). The dataset comprises a wide range of in vitro toxicity endpoints including receptor interaction, enzyme inhibition, developmental defects and cell viability. Notably, it includes the assays from the Tox21 dataset.

All three datasets are sparse, i.e., not all compounds were tested on all assays. The ToxCast dataset is the sparsest followed by the Ames dataset and then the Tox21 dataset (35.5, 40.5 and 83.4 percent of the matrix cells are filled, respectively). The ToxCast dataset has very large differences in data completeness across the assays with values ranging from 1.4% to 92.1% complete. For the Ames dataset, the assays range from 12.4% to 75.0% complete, whereas, for the Tox21 assays the percentages of filled cells range from 74.3% to 92.2%. Another important characteristic of the data is the imbalance between toxic and non-toxic labels. For the smaller datasets (see Table 1), Tox21 contains a larger imbalance of toxic vs non-toxic values compared to Ames (6.9% vs 21.3% of toxic values) with the values ranging from 3% to 15.6% for individual Tox21 assays and from 11.4% to 31.8% for individual Ames assays. For the ToxCast dataset, on average 9.4% of labels are toxic, with the percentages for individual assays ranging between 0.9% and 82.4%.

### Data processing

Chemical structures were standardized using the Python packages RDKit [23] (2019.09.03) and MolVS [24] (0.1.1). First, molecules with SMILES [25] strings from which no valid molecules could be generated were discarded. Then, bonds to metal atoms were disconnected and the charge was removed where possible. In the next step, inorganic fragments and solvents were removed. Next, certain chemotypes (e.g. nitro groups) and tautomers were transformed into a canonical form. Duplicate molecules were identified by calculating their standard InChI [26] and then transforming the InChI back to SMILES so that two molecules which share the same InChI, are guaranteed to be represented by the same SMILES. Finally, mixtures of different organic components were discarded.

At this point, the datasets contained some duplicate molecules represented by identical SMILES strings. The duplicates were aggregated to obtain a single set of data labels for each unique SMILES in the dataset. This was done by keeping the majority label (toxic or non-toxic) for each assay for identical SMILES. No data label (i.e., a data gap) was assigned to a SMILES-assay pair if toxic and non-toxic labels were of equal number. For each molecule, Morgan fingerprints of radius 2 (equivalent to ECFP4 [27]) hashed to 2048 bits were calculated with RDKit and were used as the chemical features in the models.

For the ToxCast dataset, assay filtering was performed to remove assays having insufficient data labels for robust model training and evaluation. Following chemical standardization and aggregation, only assays with at least 50 toxic and 50 non-toxic labels were kept, which reduced the size of the dataset from 617 to 416 assays and from 8615 to 7787 compounds.

### Data split into training and testing

Each dataset was split into a training set and a test set. The training sets were used to optimize hyperparameters and train the final models, and the test sets were used to evaluate the performance of the final models. Two different splitting methods were employed: compound-based and assay-based splits.

**Fig. 1:**
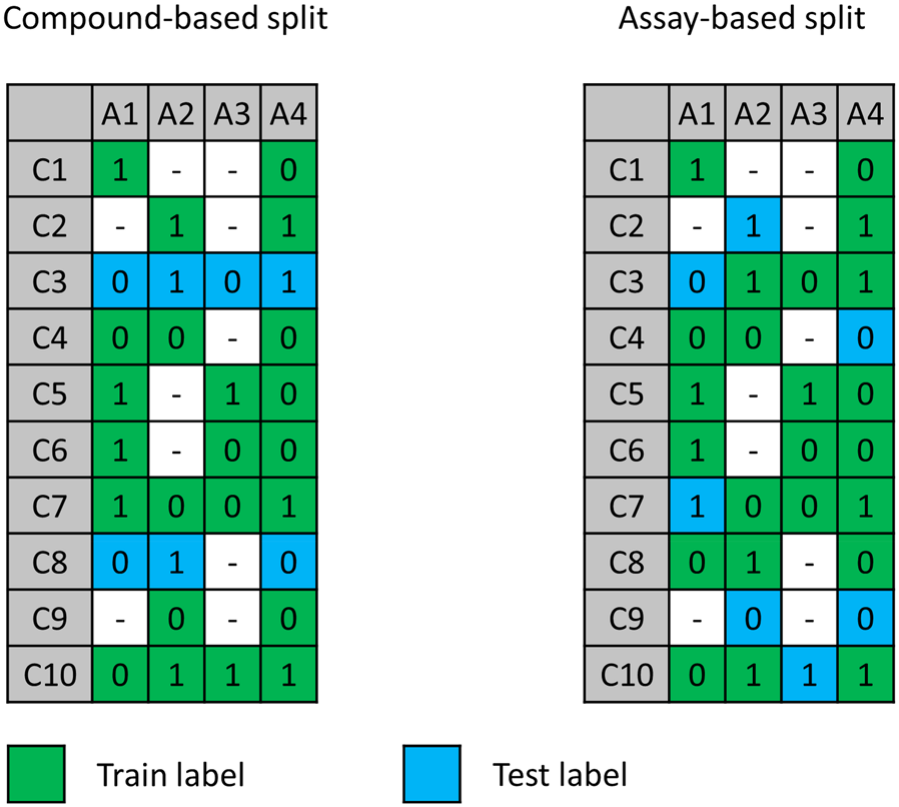
Comparison of data splitting strategies. For the compound-based split, 20% of the compounds were randomly selected for the test set. These compounds were used for testing across all the assays. For the assay-based split, each assay was considered independently and 20% of the compounds were selected at random and placed in the test set. This resulted in a different set of test compounds for each assay. *A* assay, *C* compound, *0* non-toxic, *1* toxic

The compound-based splits were used to compare the performance of multi-task models with single task models. This scenario was only tested on the Ames and Tox21 datasets due to the manageable numbers of assays. 20% of the compounds in each dataset (Ames or Tox21) were selected at random for the test set and the remaining 80% formed the training set (Fig. 1). This means that the training and test sets are the same for all the assays in each dataset.

Compound-based splits represent the established way to assess the performance of QSAR models and is the approach used, for example, in the Tox21 QSAR modeling challenge. However, the imputation techniques describe above, namely Alchemite and pQSAR, have been evaluated primarily in a different scenario, appropriate for sparse activity matrices. In this scenario, the imputation model is created using training data and applied to fill the missing values in the activity matrix. This scenario can be considered a form of repurposing, where imputation is used to search for novel activities among compounds already present in sparse databases. We implemented this testing scenario with what we call the assay-based splits (Fig. 1). Each assay was considered independently and 20% of the compounds for which the toxicity labels are known were selected at random and placed in the test set with the remaining compounds being added to the training set. Some compounds therefore appear in both the training data and the test data, however, the assay labels are split so that none of the same compound/assay label pairs appear in both the training and the test data. Thus, a compound may be in the test set for assay A, but in the training set for assay B with the toxicity label of the compound from assay B given as input to the imputation model. Due to the low number of overall labels for some of the assays, the splitting for the ToxCast dataset was done in a stratified manner (according to toxicity labels).

Only assay-based splits were used for the ToxCast dataset since the aim was to validate and extend the findings of the assay-based experiments carried out on the smaller Ames and Tox21 datasets.

### Single task models

Various single task and multi-task techniques were used to train models as described below. The hyperparameter optimization was only applied for models on the smaller datasets (Ames and Tox21). For the ToxCast dataset, identical hyperparameters were selected for all assays. These were values that occurred frequently across the assays following hyperparameter optimization on the Ames and Tox21 datasets.

#### Random Forest

Random forest (RF) is a well-established algorithm for QSAR modelling that has achieved good performance in a variety of tasks [28]. RF is an ensemble method, where different decision trees are trained on different bootstrapped subsets of the training data and different random subsets of features. For this work, the implementation of RF in the Python [29] scikit-learn [30] package (0.22.1) was used. The hyperparameters considered for optimization in the grid search are given in the Supporting Information (Additional file 1: Table S1).

#### XGBoost

Another ensemble technique is so-called boosting, which generally refers to the sequential combination of several weak (i.e., perform slightly better than random) learners in order to learn from the mistakes of previous learners [31]. In gradient tree boosting, decision trees are learned sequentially to predict the residuals (i.e., mistakes) of the previous tree, and eventually the predictions of all single trees are combined [32]. XGBoost (XGB) is a popular open-source implementation of gradient tree boosting which scales well to very large datasets [33]. The Python package XGBoost (1.0.1) was used in this study. The hyperparameters considered for optimization in the grid search are given in the Supporting Information (Additional file 1: Table S2).

#### Deep Neural Network

Deep Neural Networks (DNN) are combinations of artificial neurons organized in layers [34]. Each layer can be considered a simple non-linear machine learning model, but they can become increasingly complex by adding additional layers. The DNNs used in this project are feed forward ANNs using the Python package Tensorflow [35] (2.1.0) with the Keras API [36]. 2048 nodes were used in the input layer (one node for each bit of the chemical fingerprint) and the output layer consisted of a single node to which the sigmoid function was applied to obtain a binary output for classification (using 0.5 as the classification threshold). The DNNs for different assays contain between one and four hidden layers (number of hidden layers is a hyperparameter considered for optimization). The ReLU activation function was applied to all nodes in the hidden layers. Binary cross-entropy was used as the loss function and the Adam algorithm was used for fitting the network’s weights and biases. The hyperparameters considered for optimization are given in the Supporting Information (Additional file 1: Table S3).

### Multi-task models

#### Multi-task Deep Neural Network

As for the single task DNN models, the multi-task DNN models were trained using the Python package Tensorflow with the Keras API. A multi-task DNN is trained to predict several assays at the same time with the output layer of the network consisting of one node for each task. The sigmoid function was applied to each node in the output layer and the obtained output was binarized (using 0.5 as the classification threshold) to obtain a predicted class for each assay. The loss for a single data point (a single compound with up to 12 assay labels for Ames and Tox21 dataset) during training is its binary cross-entropy averaged across all assays. For partially complete data points (i.e., molecules for which the label is known only for some of the assays) the assays without available labels were excluded when the loss was computed. This enables training on sparse datasets. The DNN was trained using the 2048 chemical features as inputs, as for the single task models. When the multi-task model was used for prediction, a test compound was input as chemical features and the output was a vector of predicted values, one for each assay.

For the assay-based split, the multi-task DNN was trained on all compounds assigned to the training set (all of which had at least one assay label as shown in the assay-split in Fig. 1) and, for a given compound, only the labels that were assigned to the training set (green cells of the matrix in Fig. 1) were used to compute the loss during training.

#### Macau

Macau is a Bayesian probabilistic matrix factorization technique that is able to analyze sparse matrices. Probabilistic matrix factorization gained attention for recommender systems, following the 2009 Netflix competition where it was used to make predictions using the data matrix only, i.e., the ratings for viewer-movie pairs. The Macau method has recently been used for multi-task modelling in QSAR [4] and is also able to use descriptors of the entities being analysed, which are referred to as side information [5]. In the Macau models developed here, the sparse data matrix consists of compound-assay label pairs and Morgan fingerprints are used as side information for the compounds, with no side information being used for the assays. The hyperparameters considered for optimization are given in the Supporting Information (Additional file 1: Table S4). The Python package Macau (version 0.5.2) was used in this study.

#### Feature Net

**Fig. 2:**
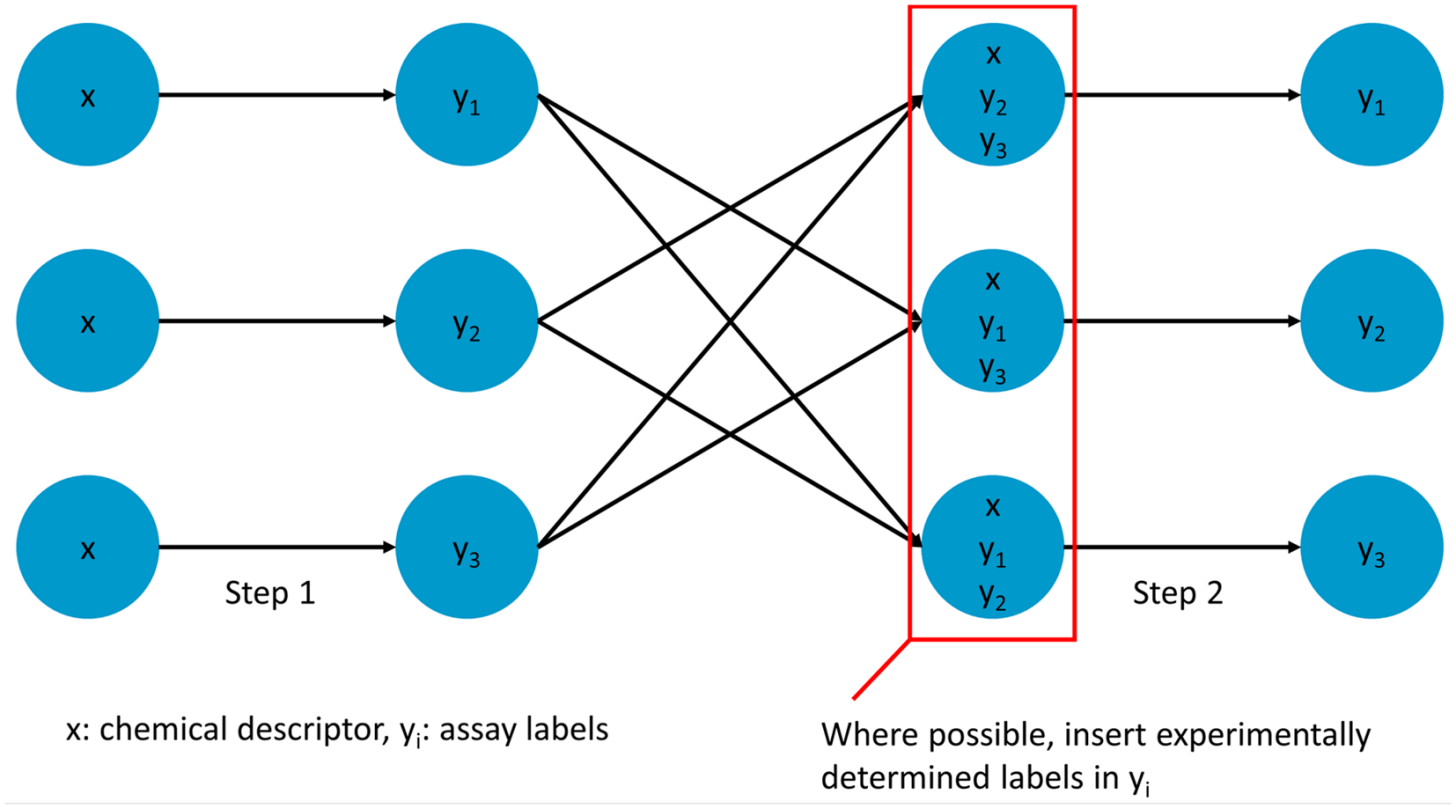
Schematic depiction of Feature Net (FN) models. In Step 1, a single task QSAR model is trained to predict the labels of missing values for each toxicity assay in turn (y_1_, y_2_, y_3_). In Step 2, the models are retrained using both chemical descriptors (x) and auxiliary assay labels as features. The labels for the auxiliary assay are either experimentally determined assay labels or the predictions from Step 1 where experimental values are not available

The Feature Net (FN) [37] technique combines multiple single task predictors into a net-like structure and can be used with any supervised machine learning algorithm. In the first step, a single task model was trained for each assay separately using the chemical features and the models were used to predict the unknown toxicity labels for the whole dataset. In the second step, the final model for each assay was obtained by retraining the single task model using the labels of the other assays explicitly as features, in addition to the chemical features. In this setting, each modelled assay is called the target assay and the other assays, used as features, are called auxiliary assays. For a given target assay, the second model is trained using compounds with experimental labels for this assay (i.e., no predicted labels), however, the labels of the auxiliary assays are either experimentally determined labels, or the predicted labels from the models in the first step, when no experimentally determined label is available. A schematic depiction of the FN models is given in Fig. 2. The FN models were trained using all of the single task methods described above using the same hyperparameters as for the single task models, that is, no additional optimization was performed.

When a FN model is used for prediction, each assay value is predicted sequentially with the test compound input as chemical features and assay labels for all other assays, that is, the auxiliary labels. The auxiliary assay labels can be predicted values or experimental values. We investigated two different scenarios that mimic how QSAR is used in practice. One common application is making predictions for virtual compounds, that is, compounds which have not yet been synthesized. In this case, we discarded any experimental values so that all the input labels for the test compounds were predicted values. This application was tested in the compound-based splits. The second application is the prediction of assay outcomes for compounds that have already been characterized for some toxicity endpoints. To simulate this case, we used experimental values for the auxiliary assays in the test set, when these were available, otherwise we used predicted values as determined in the first step of the FN method. This application was tested both in the compound-based splits (with experimental test labels) and in the assay-based splits.

### Model training

For each technique, a fivefold cross validation grid search was performed on the training set for hyperparameter optimization, except for Macau, where the grid search was performed using a single validation set containing 20% of the labels in the training set for each assay. This difference is due to the assay-based splits better reflecting the typical use of Macau and the complexity of setting up cross validation in this scenario. Furthermore, preliminary experimentation suggested that Macau is relatively insensitive to the specific hyperparameter settings. All of the models used Morgan fingerprints hashed to 2048 bits as chemical features, generated using the Python package RDKit.

### Evaluation of models

The overall performances of the models were evaluated first. Following this, the characteristics of the datasets were investigated in order to gain insights on when imputation is likely to lead to the most benefit. The different evaluation measures are described below.

#### Sensitivity to random seed

All the modelling techniques used in this study involve stochastic processes, e.g., the random initialization of weights in the neural networks or the generation of bootstrap samples in the RF models. The random behavior of these methods can be made reproducible by setting a random seed, however, the result will represent only one from a distribution of potential results. For a rigorous comparison of the different techniques, all of the final models for the Ames and Tox21 datasets were trained using 20 different random seeds and the range of results was examined. This follows the approach of Xu et al. in their comparison of multi-task DNNs with single task DNNs [38]. Due to its larger scale, evaluation on the ToxCast dataset was limited to one model instance resulting from a single random seed.

#### Overall performance

Matthews Correlation Coefficient (MCC) was chosen as the primary metric to evaluate model performance, due to its suitability for imbalanced datasets. In addition, the F1 scores and ROC-AUCs are reported in the code repository. When reporting the performance of a technique across different assays and different random seeds, the average of all assays for each seed was computed first, and the average across all seeds is reported.

MCC scores are sensitive to the selected classification threshold (0.5 by default) applied to the raw model output. For imbalanced datasets this threshold may be inappropriate and lead to poor MCC scores. Recently, GHOST (generalized threshold shifting procedure) was proposed as a method to automatically find an ideal decision threshold for classifier models using merely training instances [39]. In GHOST, a classifier is trained using all training examples and probability scores are determined for each training instance. N bootstrap samples are then drawn from all training instances and the optimal threshold is found for each sample by trying a range of different thresholds. The chosen decision threshold is taken as the median of the individual optimal thresholds. Two different approaches were used to find the optimal threshold for a bootstrap sample. In this project, we used the method originally described elsewhere [40], which determines the point of the ROC curve closest to the upper left corner (0,1). The GHOST method was applied on the models trained on the ToxCast dataset and, as in the original paper, the thresholds considered were: 0.05, 0.1, 0.15, 0.2, 0.25, 0.3, 0.35, 0.4, 0.45, 0.5, 0.55. In the multi-task settings, thresholds were optimized for each task separately.

#### Dataset characteristics

Further analyses were carried out to gain an understanding of the characteristics that lead to performance gains when using imputation techniques. These included investigations of the role of chemical similarity, the amount of experimental data available, and correlations between assays.

For the chemical similarity experiments (conducted for the Ames and Tox21 dataset), the test sets used in the assay-based splits were divided into three bins, depending on the chemical similarity of each test compound to the training compounds of the respective assay. Chemical similarity was evaluated as the average Tanimoto similarity to the five nearest neighbors in the training set based on Morgan fingerprints and bins were defined with similarity ranges: 0–0.4, 0.4–0.6, 0.6–1. Results are reported for assays with at least 100 test compounds in each of the three bins. The performance of each model was evaluated on each of the bins independently and, in each case, the results were averaged over the 20 models obtained using different random seeds.

The impact of data sparsity was investigated in two different ways. For the Ames dataset, sparsity in terms of available experimental labels for test compounds was measured. The test sets used in the assay-based splits were divided into three bins according to the number of experimentally determined toxicity labels for each test compound in the training set data. The bins were: 0–1 labels, 2–3 labels; and > 3 labels. Results are reported for assays with at least 100 test compounds in each of the three bins. The performance of each model was evaluated on each of the bins independently and, in each case, the results were averaged over the 20 models obtained using different random seeds. This analysis was not possible for the Tox21 dataset, as this dataset contains very few compounds that were tested in only a few of the assays.

An investigation of the effect of overall dataset sparsity on imputation model performance was conducted on the ToxCast dataset. In particular, sparsity in the training set was artificially increased by randomly sampling 1000 labels per assay for model training with all other labels removed. For assays with fewer labels in the training set, all labels were kept. The overall completeness of the training set was reduced from 29.4% to 10.9% in this scheme. Models were evaluated on the same test set as for the models trained using all available labels to enable comparison of model performances.

To examine the importance of single assays on the overall success of FN models, pairwise FN models were trained, where just one auxiliary assay was used as an additional feature. For each target assay, the remaining auxiliary assays were tested in turn in pairwise FN models and the scores of the models were compared to the score of a single task QSAR model for the target assay. Similar to the full FN models and the single task QSAR models, each pairwise FN model was trained using 20 different random seeds and the median MCC score across the 20 models was used to represent each particular model. A large improvement in performance would suggest a high importance of the respective auxiliary assay for the full FN model.

The importance of a single auxiliary assay to the overall success of the full FN model was also examined using information theory [41]. The entropy of an assay is computed as:

$$H\left(A\right)= - {(P}_{A}\times {\text{log}}_{2}{P}_{A}+ {P}_{a}\times {\text{log}}_{2}{P}_{a})$$

where *P*_*A*_ is the probability of a randomly selected compound being active in the assay, computed by the ratio of compounds with the label ‘toxic’ to all labelled compounds, and *P*_*a*_ the proportion of non-toxic compounds, given by 1-P_A_. The mutual information of two assays is computed by taking the sum of the entropies of each assay and subtracting the entropy of the joint distribution:

$$MI\left(A,B\right)=H\left(A\right)+H\left(B\right)-H(A,B)$$

The entropy of the joint distribution is computed as:

$$H\left(A,B\right)=-({P}_{AB}\times {\text{log}}_{2}{P}_{AB}+{P}_{Ab}\times {\text{log}}_{2}{P}_{Ab}+{P}_{aB}\times {\text{log}}_{2}{P}_{aB}+{P}_{ab}\times {\text{log}}_{2}{P}_{ab})$$

where *P*_*AB*_ is the proportion of compounds labelled as toxic in both assay A and B, *P*_*ab*_ is the proportion of compounds that are non-toxic in both assay A and assay B, and *P*_*Ab*_ and *P*_*aB*_ are the proportions of compounds toxic only in assay A or only in assay B, respectively. The proportions were only computed for compounds that are labelled for both assay A and assay B.

The relatedness between a target assay and an auxiliary assay was measured by the ratio of mutual information (MI) between two assays to the entropy of the target assay. This measure of relatedness, called here MI-entropy ratio, describes how much of the total entropy (or amount of information) of the target assay (here assay A) is contained in the auxiliary assay.

$$MI-entropy\; ratio\; (A,B)= \frac{MI(A,B)}{H(A)}$$

Observed differences of pairwise FN models to the respective single task models were analyzed with respect to the MI-entropy ratio of the involved assay pair. Pairwise FN models were only obtained for the Ames and Tox21 datasets and this full analysis was not conducted on the larger ToxCast dataset due to the much larger number of assays. However, the usefulness of the MI-entropy ratio to select auxiliary assays from a large dataset was tested using one exemplary target assay from the ToxCast dataset. This is the assay ‘TOX21-Aromatase-Inhibition’ as it showed a large increase in performance when using multi-task imputation models compared to single task models. In addition, the assay possesses a relatively large number of experimental labels in the test set (1431 in total with 221 of these toxic:), which enables a robust evaluation of model performance. Increasing numbers of auxiliary assays (1, 3, 5, 10, 20) were selected either randomly in an additive approach (i.e., the 3 sampled auxiliary assays include the assay that was sampled for 1 auxiliary assay, and so on) or according to the MI-entropy ratio in descending order. For this experiment, 20 different random seeds were used during model training for a more robust evaluation.

## Results

### Traditional single task and multi-task models

In the first step, single task and multi-task models were compared using traditional compound-based train-test splits. The performances of the single task and multi-task models are reported in Table 2 as median (and interquartile range) MCC scores over the 20 independent runs for each assay using different random seeds. FN models were trained using RF, XGB and DNN but only XGB-FN is shown as this was the best performing method. Of the single task methods, XGB provided the best average MCC score for both the Ames and the Tox21 dataset, albeit the differences were quite small. The multi-task methods showed reduced performance when averaged over the Ames assays. The best multi-task method for the Ames dataset was multi-task DNN which performed better than RF and single task DNN, however, it was worse than the single task XGB. Overall, while the differences are quite small, this shows that multi-task methods are not guaranteed to improve on single task performance. For the Tox21 assays, the FN models and multi-task DNN provided a slight benefit over the single task models. The Macau technique was outperformed by the other methods on both datasets based on MCC with this difference being more pronounced for the Tox21 data compared to the Ames data.

**Table 2:** Median MCC scores of single task and multi-task QSAR models

		Ames	Tox21
Single task	RF	0.526 (0.523–0.527)	0.402 (0.400–0.405)
	XGB	**0.547** (0.545–0.550)	0.427 (0.422–0.430)
	ST-DNN	0.519 (0.508–0.527)	0.417 (0.410–0.426)
Multi-task	XGB-FN	0.529 (0.527–0.531)	**0.435** (0.432–0.438)
	MT-DNN	0.540 (0.527–0.549)	0.430 (0.423–0.437)
	Macau	0.490 (0.484–0.499)	0.321 (0.319–0.323)

Median MCC scores and interquartile range for each technique and dataset on the test set across the 20 random seeds. Before computing the median, the mean across the different assays for a single run was calculated. The best model for each dataset in bold

The relative performances using ROC-AUC are shown in Additional file 1: Table S5. Both multi-task DNN and Macau outperformed the single task approaches on both the datasets using this metric. For the FN models, no consistent improvements were found for the multi-task methods over the single task models for either metric.

**Fig. 3:**
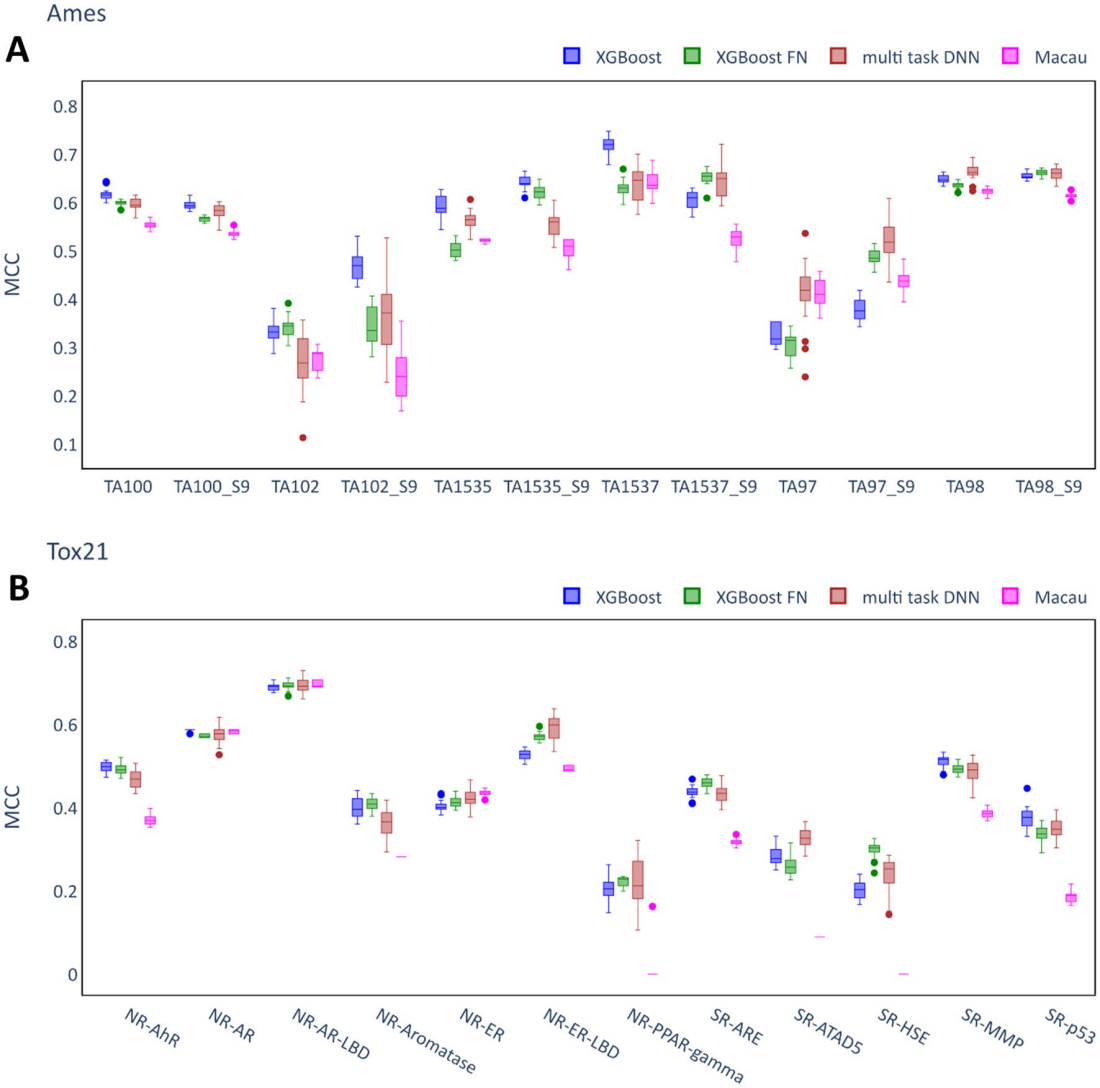
Performance of multi-task QSAR models. **A** Ames dataset. **B** Tox21 dataset. Each box summarizes the MCC scores of 20 independent runs of the model on the test set with identical hyperparameters but differing random seeds. The XGB models (best performing single task QSAR model) are shown as a baseline model. Only the best performing Feature Net model (XGB-FN) is included.

Figure 3 compares the performances of the multi-task models to XGB as the best single task model for the individual assays where it can be seen that single task models performed better for some assays whereas multi-task models performed better for others. Therefore, although the multi-task models were beneficial for some assays, they did not outperform single task models consistently in these datasets.

It is notable that the variance between different runs (with identical hyperparameters but different random seeds) was quite large in some cases. Among the multi-task techniques, multi-task DNN showed the largest variances. In addition, it appears that regardless of model type some assays are more prone to large variance than others, for example, TA102 (Fig. 3A) and NR-PPAR-gamma (Fig. 3B). Notably, the variance is consistently smaller when evaluating the models using ROC-AUC as metric (see Additional file 1: Fig. S1). While the ROC-AUC metric evaluates the capability to rank compounds according to their probability of being toxic, the MCC metric is sensitive to the classification of compounds based on a decision threshold. This may have caused the relatively large variances in MCC scores, especially on very imbalanced assays.

**Fig. 4:**
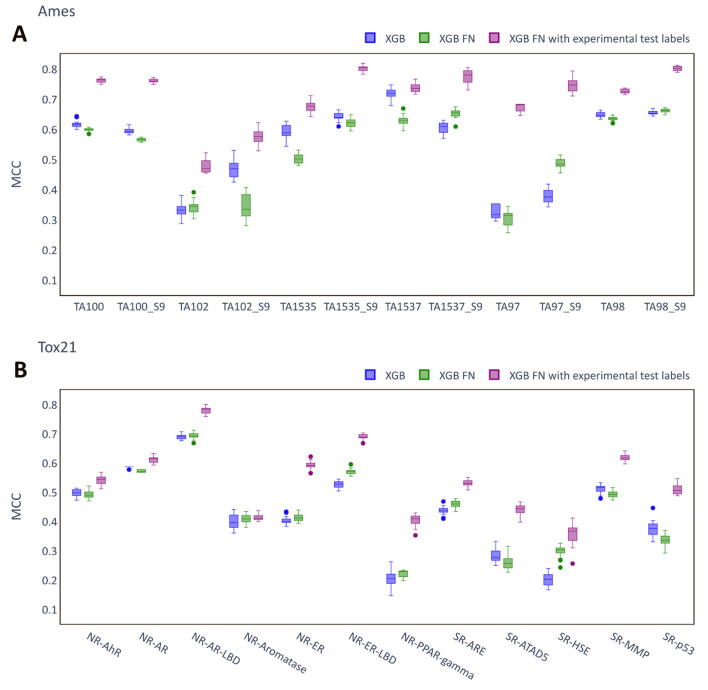
Performance of FN models. **A** Ames. **B** Tox21. The plots compare the MCC scores of XGB-FN models to single task XGB models. The first FN model in each plot corresponds to the situation where predicted values are used for the labels of the auxiliary assays of test set compounds, whereas in the second case experimental labels are used in place of the predicted values for the auxiliary assays, when these are available. Each box summarizes the MCC scores of 20 independent runs of the model on the test set with identical hyperparameters but different random seeds.

As mentioned in the methods, the FN models use auxiliary assay values as inputs. In the results presented above, the auxiliary values used for the test sets were predicted values. This simulates the case when no experimental values are available for the compounds for which predictions are made. The performances of the FNs were re-evaluated by using experimental values for the test set compounds, where these were available, in place of the predicted values. Figure 4 reports the MCC scores for these models (called FN with experimental test labels) compared to the single task models and the FN with predicted test labels for the Ames and the Tox21 dataset, respectively. The FN models with experimental test labels outperformed both the single task and the FN models with predicted test labels consistently across all the assays and methods, in many cases by a wide margin (e.g. TA97 in Fig. 4A). The only two exceptions were NR-AR and NR-Aromatase (Fig. 4B), where there was no benefit. Generally, the increases in performance were larger for the Ames dataset compared to the Tox21 dataset (Table 3). For instance, the RF-FN models provided an average MCC score exceeding the single task RF by nearly 0.2 (0.723 vs. 0.528), whereas the corresponding difference for the Tox21 dataset was smaller than 0.1 (0.490 vs. 0.402). Overall, RF-FN performed best on the Ames dataset, while XGB-FN achieved the highest average score on the Tox21 dataset. The FN models using experimental test labels as inputs clearly outperformed both single task and conventional multi-task QSAR models.

**Table 3:** Median MCC scores of Feature Net models

		Ames	Tox21
RF	Single task	0.526 (0.523–0.527)	0.402 (0.400–0.405)
	FN with predicted test labels	0.509 (0.507–0.513)	0.376 (0.374–0.378)
	FN with experimental test labels	**0.723** (0.721–0.726)	**0.490** (0.487–0.494)
XGB	Single task	0.547 (0.545–0.550)	0.427 (0.422–0.430)
	FN with predicted test labels	0.529 (0.527–0.531)	0.435 (0.432–0.438)
	FN with experimental test labels	**0.710** (0.704–0.713)	**0.541** (0.538–0.543)
DNN	Single task	0.519 (0.508–0.527)	0.417 (0.410–0.426)
	FN with predicted test labels	0.526 (0.516–0.529)	0.408 (0.397–0.413)
	FN with experimental test labels	**0.677** (0.675–0.682)	**0.500** (0.488–0.504)

Median MCC scores and interquartile range for each technique and dataset on the test set across the 20 random seeds. Before computing the median, the mean across the different assays for a single run was calculated. The best model of each base algorithm (RF, XGB and DNN) for each dataset is in bold.

### Single task and multi-task imputation models

The assay-based data splits were used to investigate the underlying reasons for the improved performance of the FN models with experimental test labels, following a similar approach reported by Martin et al. and Whitehead et al. in their studies on imputation [10, 11]. Here, this approach is referred to as assay-based splits and resembles the challenge of filling gaps in a sparse matrix (see Fig. 1). Thus, for a given compound, some of the assay labels may be assigned to the training set, while others may be assigned to the test set. When the FN model predicts the label of a particular assay-compound pair, other assay labels for that compound may have been used as training data in addition to that compound’s chemical features. In contrast to the studies by Martin et al. and Whitehead et al., a random split of the data was made instead of a cluster-based split, as this enabled the performance of the different imputation models on test compounds with low, medium and high chemical similarity to compounds in the training set to be investigated separately.

**Fig. 5:**
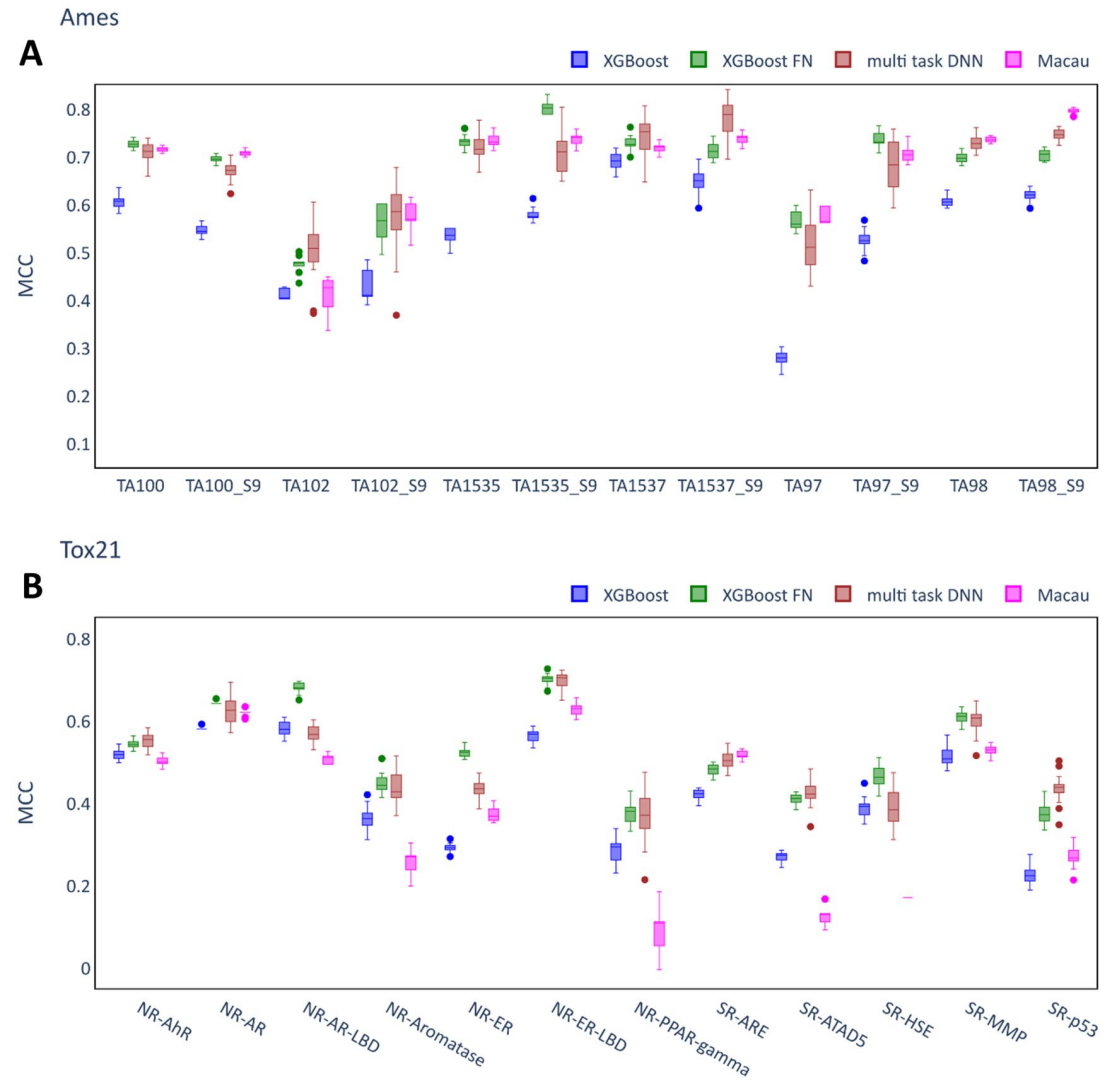
Performance of imputation models. **A** Ames dataset. **B** Tox21 dataset. Each box summarizes the MCC scores of 20 independent runs of the model on the test set with identical hyperparameters but different random seeds. The XGB models (best performing single task model) are included as a benchmark. Only the best performing Feature Net model (XGB-FN) is included in this plot.

Figure 5 compares the MCC scores for the FN models, the multi-task DNN models and Macau models on the Ames and the Tox21 datasets. As before, although the FNs were trained using XGB, RF and DNN, only the best performing FN model (XGB-FN) is included. The results of the best single task model (XGB) on the assay-based splits are included as baseline models.

All of the multi-task methods outperformed the XGB single task models on the Ames dataset (Fig. 5A). In fact, the margin between the XGB models and all of the multi-task models was remarkably large (more than 0.1 difference in median MCC scores) for many of the assays (e.g. TA97, TA1535). Exceptions were TA102 and TA1537, where the benefits of the multi-task methods were comparatively small. Table 4 reports the median MCC scores for the different imputation techniques across different random seeds for both datasets. Macau achieved the highest average score on the Ames dataset (0.679), however, the differences compared to XGB-FN (0.677) and multi-task DNN (0.676) were marginal.

**Table 4:** Median MCC scores of imputation models

		Ames	Tox21
Single task	RF	0.520 (0.517–0.523)	0.406 (0.404–0.412)
	XGB	0.540 (0.537–0.543)	0.415 (0.412–0.421)
	ST-DNN	0.500 (0.495–0.507)	0.415 (0.406–0.419)
Multi-task	XGB-FN	0.677 (0.675–0.682)	**0.521** (0.516–0.525)
	MT-DNN	0.676 (0.667–0.688)	0.503 (0.493–0.512)
	Macau	**0.679** (0.677–0.681)	0.385 (0.379–0.388)

Single task models are included as a benchmark. Median scores and interquartile ranges for each technique and dataset on the test set across 20 different random seeds. Before computing the median, the mean across the different assays for a single run was calculated. The best model for each dataset is in bold.

For the Tox21 dataset, the highest average MCC scores were achieved for XGB-FN (0.521) and multi-task DNN (0.503), however, the benefits of the multi-task methods were smaller compared to the Ames dataset. There were a few cases where the difference in median MCC score between the FN model and the XGB models was above 0.1 (e.g. XGB-FN for NR-ER). Overall, in contrast to the Ames dataset, Macau performed worse than the single task models. Consistent with the findings in the previous section, the multi-task DNN models showed a comparatively large variability in performance across different runs of a particular model.

**Fig. 6:**
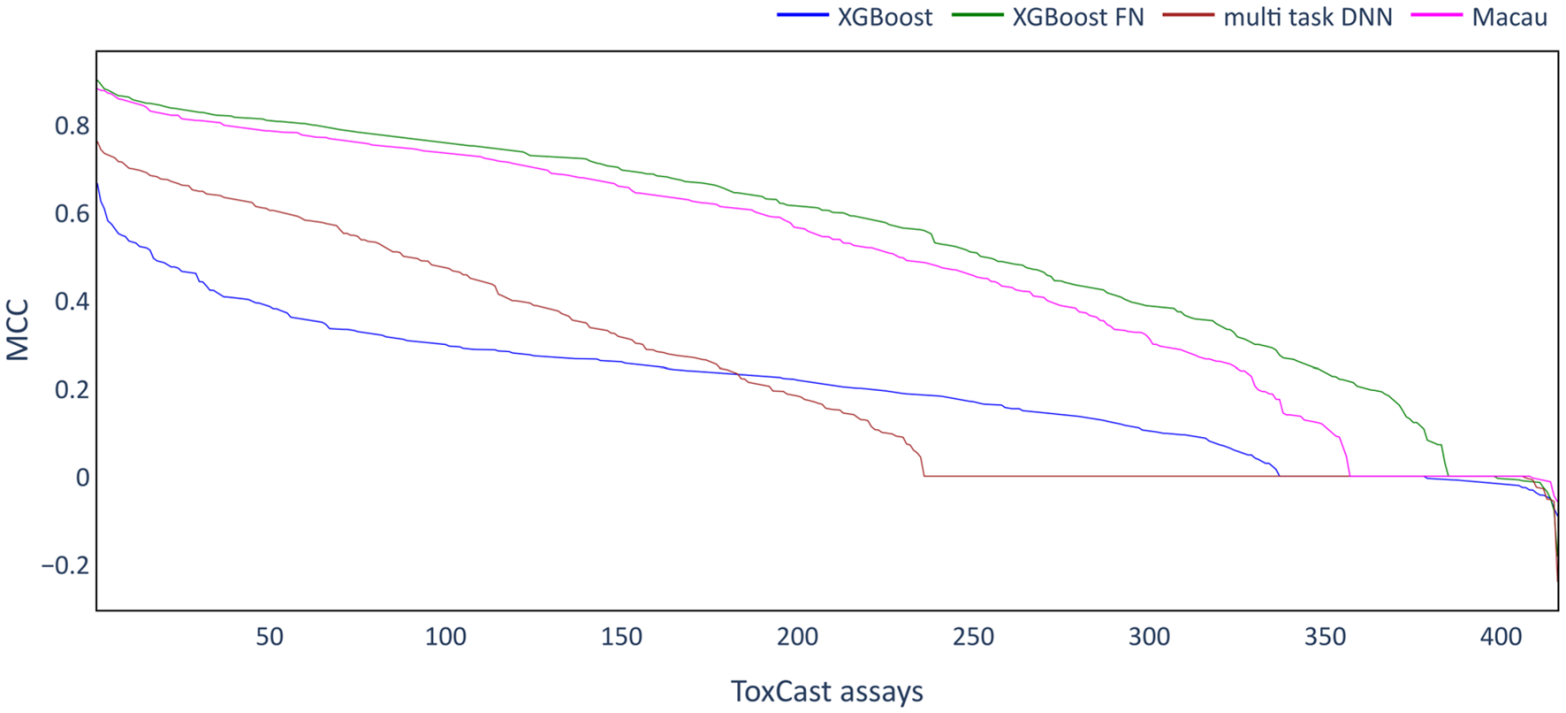
Performance of the imputation models on the ToxCast dataset. MCC scores obtained for test set of the individual assays are sorted in descending order and plotted as a line.

The imputation experiments were then extended to the much larger ToxCast dataset consisting of 416 toxicity assays (after preprocessing) to investigate the generality of the results obtained on the Ames and Tox21 datasets. Figure 6 shows the MCC scores of the different multi-task imputation models together with XGB as a single task technique. For each technique, the assays are sorted on descending MCC score, as done previously for pQSAR [10].

A wide range of MCC scores is obtained for the different models and the different assays. XGB as single task approach is clearly outperformed by XGB-FN and Macau models across the whole range of assays. The multi-task DNN models outperformed XGB on some assays, but did not perform nearly as well as Macau or XGB-FN. These results confirm those found on the smaller datasets (Ames and Tox21) and show that multi-task imputation models can provide a large benefit on the ToxCast dataset, with Macau and XGB-FN models achieving MCC scores above 0.8 for some of the assays. While XGB-FN models achieved higher MCC scores than Macau, observed ROC-AUC scores are very similar (Additional file 1: Fig. S2).

**Fig. 7:**
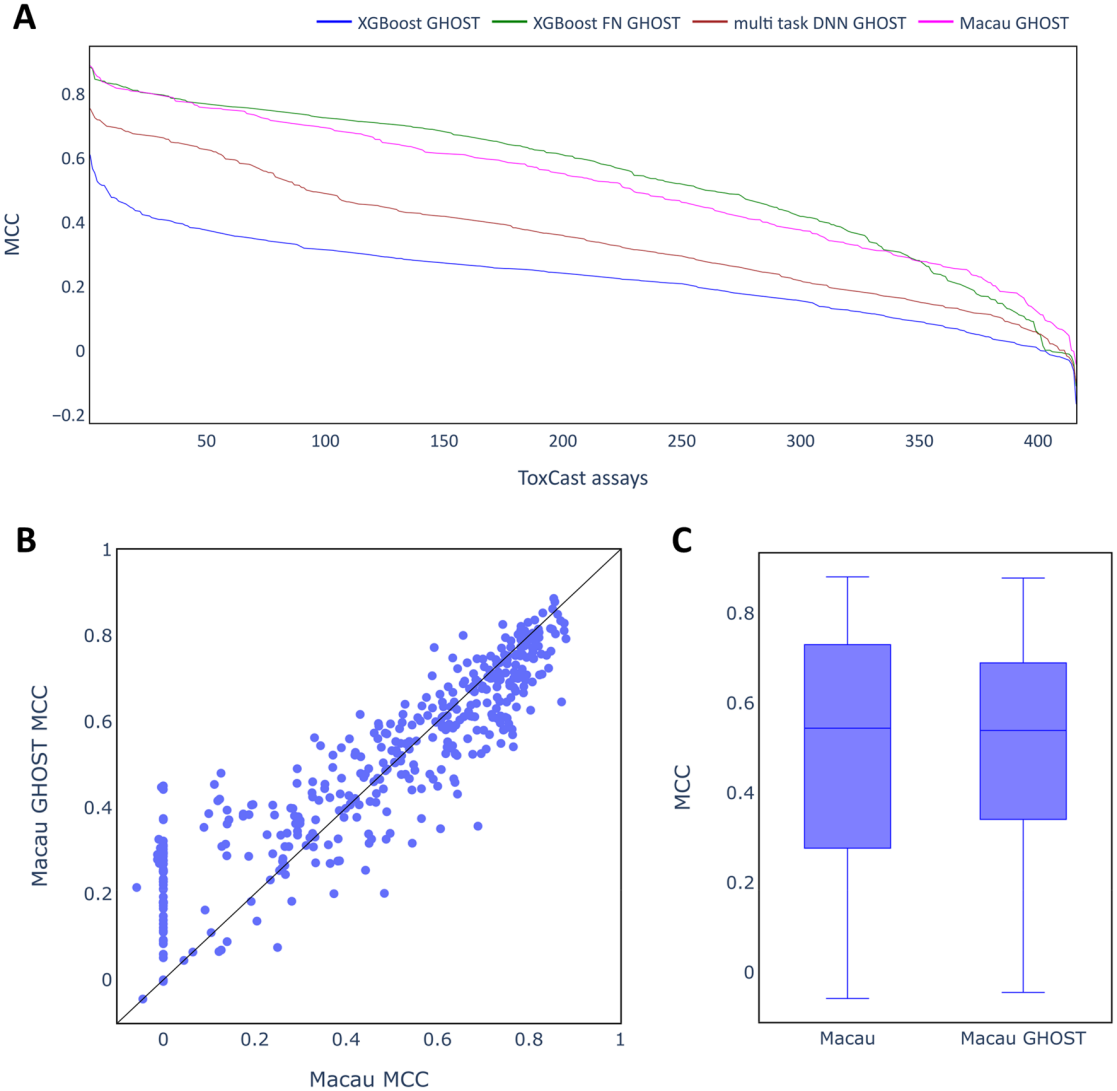
Performance of the imputation models using the GHOST approach. **A** line plots showing performance on the test set for all algorithms as in Fig. 6. **B** scatter plot contrasting MCC scores of Macau model for all individual assays. **C** box plots contrasting distribution of MCC scores for all assays.

It is notable that MCC scores of 0 or even below were found for some of the assays for all of the modelling methods, with this occurring most frequently for multi-task DNN models where more than 150 assays had very low scores. Very low MCC scores may be the result of an inappropriate classification threshold and the GHOST approach [39] was used to investigate the impact of adjusting the classification threshold on MCC scores. In Fig. 7A, MCC scores based on GHOST-optimized thresholds for all models are shown, while in B the MCC scores for the individual assays with and without GHOST are shown as a scatter plot for the Macau models. Similar plots for the other modelling methods are included in the supplementary information (Additional file 1: Fig. S3). The distributions of MCC scores for Macau can be seen in Fig. 7C as box plots.

Applying the GHOST methodology had a particularly strong effect on those assays where the original models achieved MCC scores of around 0, as is shown clearly in Fig. 7B. In contrast, for assays where a score larger than 0.6 was obtained without GHOST, GHOST mainly seems to decrease the performance. Overall, the median MCC score was not improved (Fig. 7C). Similar observations can be made for the other model types (see Additional file 1: Fig. S3). It can be concluded that GHOST may be helpful to find a suitable classification threshold for poorly performing models. However, for well-performing models attempts to adjust the threshold may actually decrease the MCC scores. Since no clear improvement was observed overall, GHOST was not used in the subsequent analyses on the ToxCast dataset.

### Role of chemical similarity on imputation models

**Fig. 8:**
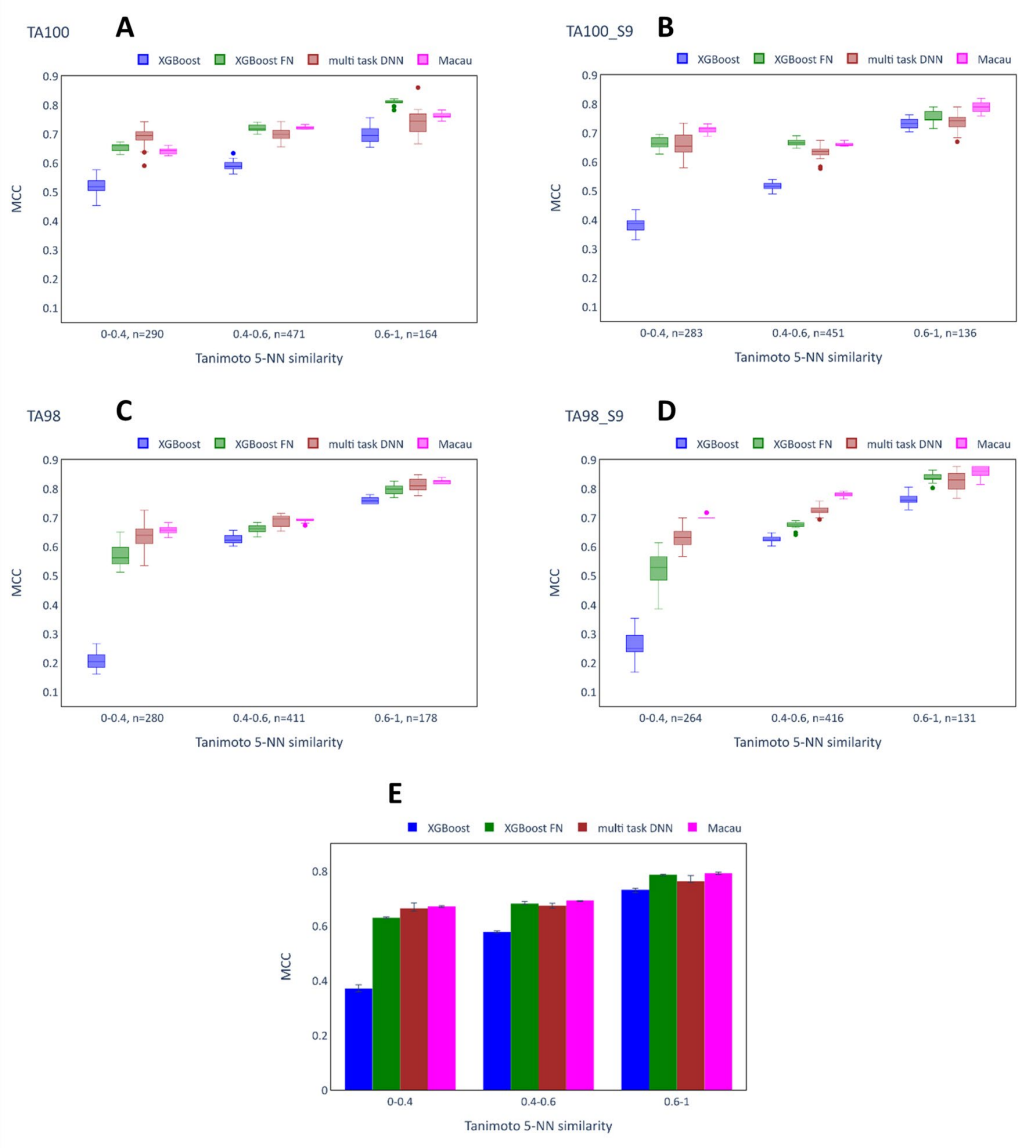
Performance of imputation models depending on test compound chemical similarity: Ames dataset. **A**–**D** show the MCC scores obtained in 20 independent runs (same hyperparameters, different random seeds) of the models on the different bins of the test set for TA100, TA100_S9, TA98 and TA98_S9. The number (n = x) written next to each bin indicates the number of compounds placed in the bin. **E** shows the median MCC scores and interquartile ranges across the different runs after computing the mean across the assays for a single run.

This section investigates the role of chemical similarity on the effectiveness of single task and multi-task imputation models based on test set predicted values reported in the previous section for the Ames and Tox21 datasets. The test compounds were assigned to similarity bins as described in the data section and the performance of the models was evaluated on each bin independently. Figure 8 shows the MCC scores for the different bins for the Ames assays TA100 (8A), TA100_S9 (8B), TA98 (8C), TA98_S9 (8D) and the average across those four assays (8E). The remaining assays had fewer than 100 compounds in each bin and were therefore excluded from this analysis. The single task XGB models achieved progressively higher scores with increasing similarity, which are in the range from 0.5 to 0.6 for the second bin (0.4–0.6 similarity) and 0.65 to 0.75 for the third bin (0.6–1 similarity). However, the performance of the XGB models was particularly poor for the most dissimilar compounds (0–0.4 bin) with, for example, median MCC scores between 0.2 and 0.3 for TA98 and TA98_S9, respectively (8C and 8D). In contrast, all of the multi-task techniques achieved much higher scores for this bin. Most notably, Macau achieved MCC scores in the range 0.65–0.7 for these two assays, but the multi-task DNNs (0.6–0.65) and XGB-FN (0.5–0.55) models also outperformed the XGB models by a wide margin. Similar trends were observed for the TA100 (8A) and the TA100_S9 (8B) assays. However, given the improved performance of the XGB models on these assays (medians around 0.4–0.5), smaller margins were observed.

The multi-task models also consistently outperformed the XGB models on the bins of more similar compounds (similarity values between 0.4–0.6 and between 0.6–1) and, similarly to the XGB models, the performance of the multi-task models tended to increase for more similar compounds. However, the margins between the single task XGB model and the multi-task models were much smaller.

These observations on the relative performance of the single task and the multi-task methods at low similarity values are supported by considering the averages across the assays in Fig. 8E. Clearly, the largest numerical benefit of the multi-task imputation techniques was found for the bin containing the most dissimilar compounds, where XGB as a conventional QSAR model performed relatively badly. Generally, the different multi-task imputation methods achieved comparable MCC scores on the different bins, with the exception that XGB-FN performed somewhat worse on the dissimilar compounds than the other techniques. These results show that the multi-task imputation models were less beneficial on the test compounds which are highly similar to compounds in the training set, but this is likely due to the higher scores overall.

**Fig. 9:**
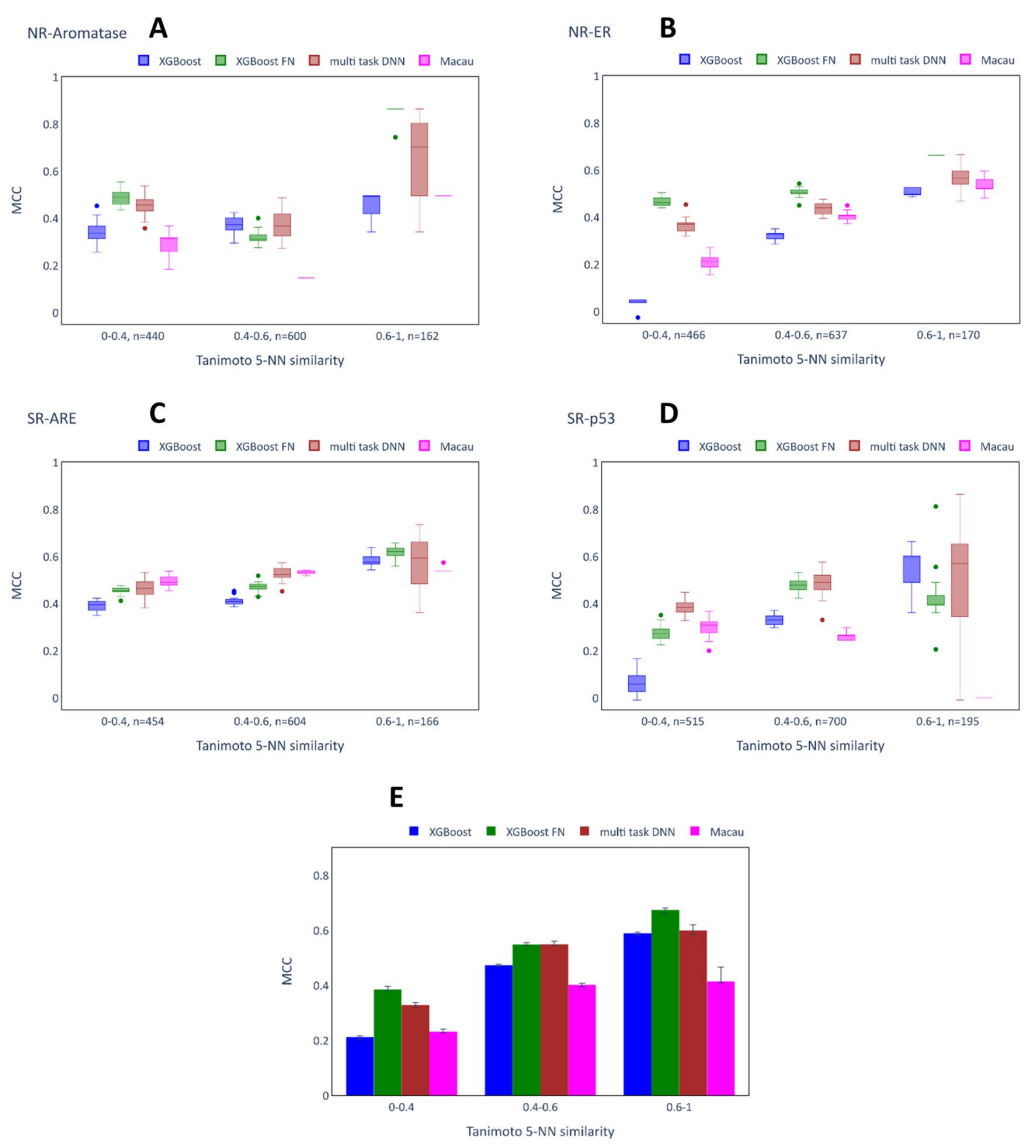
Performance of imputation models depending on test compound chemical similarity: Tox21. **A**–**D** show the MCC scores obtained in 20 independent runs (same hyperparameters, different random seeds) of the models on the different bins of the test set for NR-Aromatase, NR-ER, SR-ARE, SR-p53 (representative assays). The number (n = x) written next to each bin indicates the number of compounds placed in the bin. **E** shows the median MCC scores and interquartile ranges across the different runs after computing the mean across all the assays for a single run.

Figure 9 shows the MCC scores of four representative assays (A: NR-Aromatase, B: NR-ER, C: SR-ARE, D:SR-p53) of the Tox21 dataset for the different chemical similarity bins, as well as the average across all assays (E). Similar to the Ames dataset, the MCC scores of the XGB models were consistently higher for bins of higher chemical similarity. This effect was particularly strong for the NR-ER and the SR-p53 datasets. Likewise, the MCC scores of the multi-task models tended to increase for more similar compounds. An exception is the SR-p53 assay, where Macau achieved a MCC score of zero in the bin of highest chemical similarity. Generally, the results on this bin seem out of line, as the variability for all of the other models was very high (in the most extreme case ranging from − 0.01 to 0.864 for multi-task DNN). This is explained by the very low number of true positives in this bin (four out of 195 (2.1%), whereas the 0.4–0.6 bin contains 34 toxic compounds (4.9%) and the 0–0.4 bin contains 48 toxic compounds (9.3%)), such that small changes in predictions made by the model have a very large effect on the MCC score.

Overall, the multi-task methods (except Macau) achieved higher scores than the XGB models on the Tox21 dataset. For the assays NR-ER and SR-ARE, the largest benefit was found for the bin representing the chemically most dissimilar compounds, as was the case for the Ames dataset. For this bin and these assays, the XGB model performed particularly poorly with an MCC of around 0.1, while some multi-task models achieved MCC values up to 0.5. In the NR-Aromatase assay, both the XGB-FN (0.863) and the multi-task DNN (0.703) model achieved remarkably high median scores compared to the XGB models (0.495). However, similarly to SR-p53, the variance for the multi-task DNN was extremely large, which complicates the interpretation. When considering the average values across all assays, both XGB-FN and multi-task DNN outperformed the XGB models on all of the bins. On average, the highest benefit of the multi-task models was found for the most dissimilar compounds, but this trend is less clear compared to the Ames dataset.

### Role of data sparsity on imputation models

**Fig. 10:**
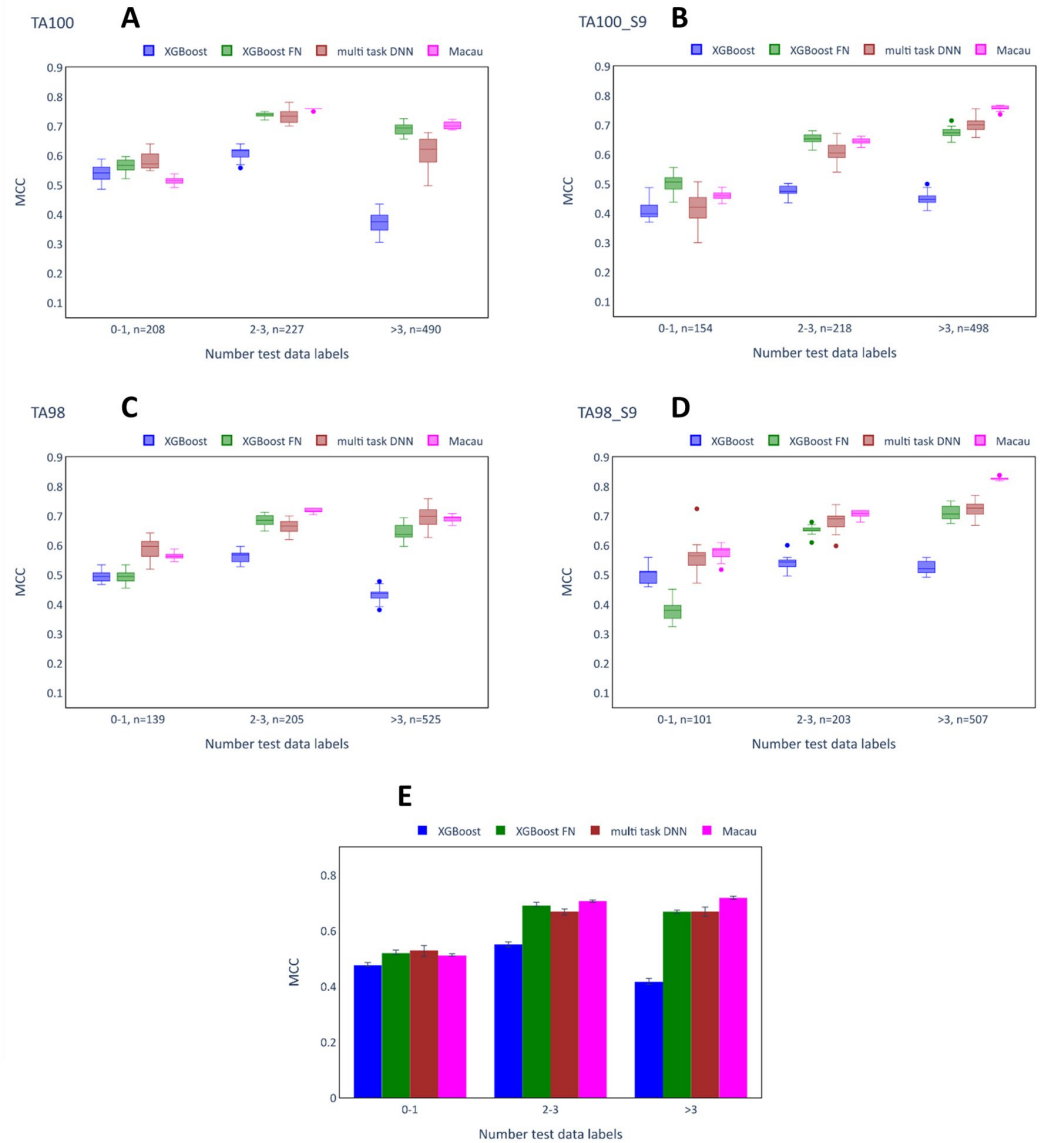
Performance of imputation models according to test compound label availability. **A**–**D** show the MCC scores obtained in 20 independent runs (same hyperparameters, different random seeds) of the models on the different bins of the test set for TA100, TA100_S9, TA98 and TA98_S9. The number (n = x) written next to each bin indicates the number of compounds placed in the bin. **E** shows the median MCC scores and interquartile ranges across the different runs after computing the mean across the assays for a single run.

The role of data availability on the effectiveness of single task and multi-task imputation models was investigated. As for the analysis on chemical similarity, the analysis was done on predicted values from the previous evaluation of single task and multi-task imputation models. The test set for each assay was divided into three bins according to the number of experimentally determined data labels (0–1, 2–3, and > 3 available labels) each compound has for the 11 remaining assays in the training set. The multi-task imputation models incorporate information about the remaining assays whereas the XGB models (single task models) do not. This analysis was only performed for the Ames dataset, as the lower sparsity of the Tox21 dataset was such that the bins of low data availability were not sufficiently populated. The analysis for the Ames dataset was limited to assays for which at least 100 compounds could be placed in each of the bins and these are the same assays as considered for the chemical similarity studies. The MCC scores for these assays (TA100, TA100_S9, TA98, TA98_S9) for the different bins and the average scores across these assays are reported in Fig. 10.

For the first bin (0–1 available labels), the MCC scores of the multi-task models tended to be only slightly higher than those for the XGB models. The multi-task DNN models were the only imputation models with higher median MCC score than the XGB model across all the assays. The other multi-task models achieved lower median scores than the XGB models for this bin in one of the assays (Macau for TA100: 0.515 vs. 0.541 and XGB-FN for TA98-S9: 0.380 vs. 0.512). For the remaining two bins, which represent a higher number of available toxicity labels for the test compounds (2–3 and > 3 available labels), the XGB models were outperformed by all of the multi-task techniques for all of the assays. The differences in MCC score between the multi-task models and the XGB models were largest for the third bin (> 3 available labels), with the highest uplift occurring for the Macau model on the TA98_S9 assay (0.829 vs. 0.520). Generally, all of the multi-task imputation models achieved similar scores for the second and third bin, but Macau performed better than the other imputation techniques for the third bin.

The observations for the single assays are supported when considering the averages across the assays as depicted in Fig. 10E. For the first bin, the XGB model was outperformed by all the multi-task models, albeit by a comparatively small margin. The margin between single task and multi-task models increased with more available data labels for test compounds. Notably, Macau outperformed the other multi-task approaches on the bin with > 3 test data labels.

This analysis shows that the number of available experimentally determined assay labels for test compounds strongly affected the performance of the imputation models. For the Ames dataset, the multi-task models clearly performed better on compounds with a high number of available assay labels.

**Fig. 11:**
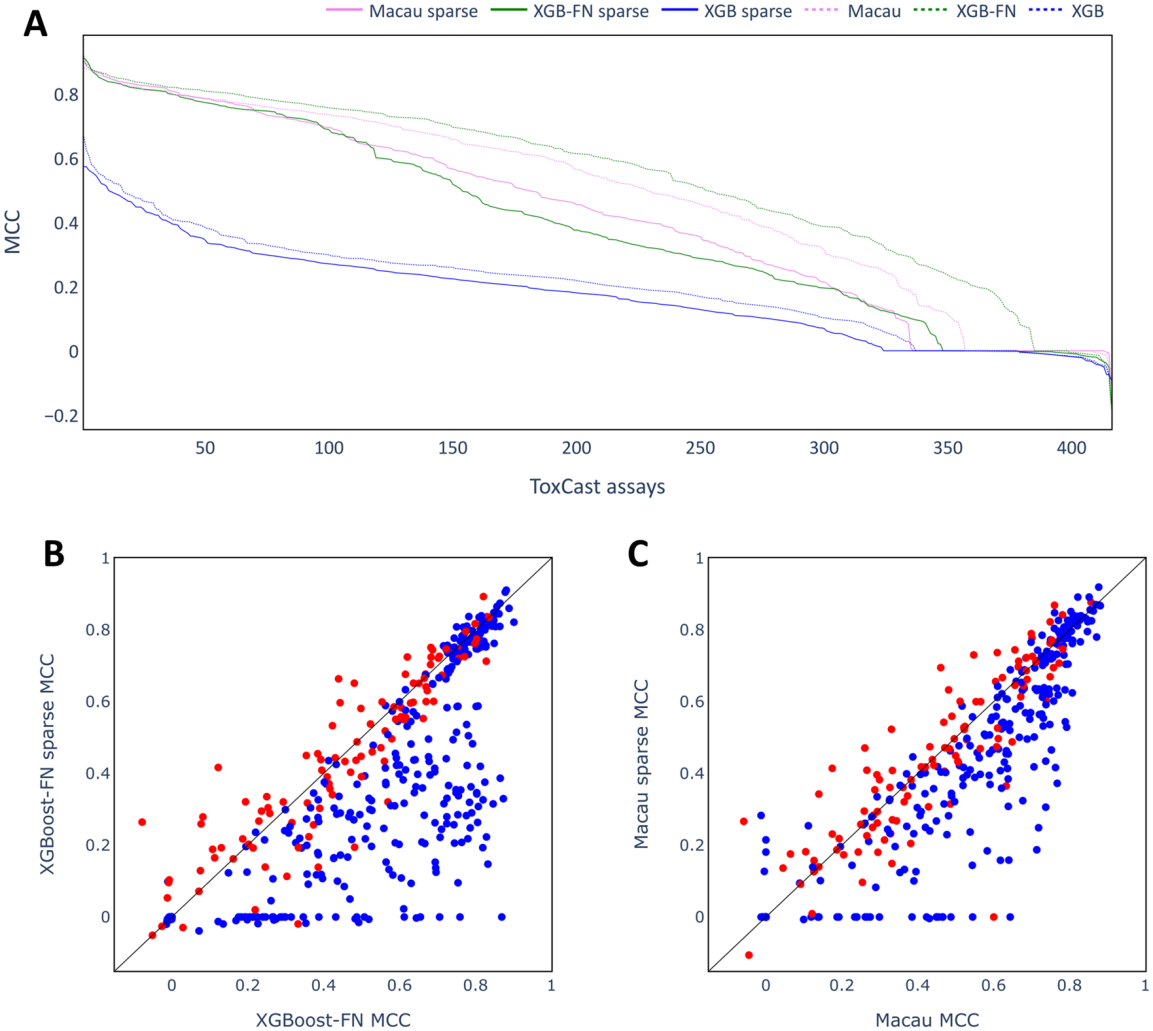
Performance of models trained on training sets of increased sparsity. **A** line plot comparing performances of different techniques on the test set. Dotted lines indicate performances of models on original datasets (sparsity not increased). **B** scatter plot contrasting MCC scores of XGB-FN model for all individual assays. **C** scatter plot contrasting MCC scores of Macau model for all individual assays. Assays whose number of training points was unchanged (≤ 1000 data points originally) are colored red, those with reduced number of training points (> 1000 data points originally) are colored blue.

The larger size of the ToxCast dataset enabled a more detailed study of the effect of sparsity on imputation. Labels in the ToxCast training set were removed to artificially increase sparsity in the dataset. As discussed above, the number of training labels was reduced to 1000 for all assays, with no changes made for assays with fewer labels in the original dataset. Figure 11A shows the performance of Macau, XGB-FN and XGB models on the dataset with increased sparsity. In Fig. 11B and c assay-wise performances are contrasted for XGB-FN and Macau, respectively, whereby assays with unchanged number of training labels (see above) are colored in red.

The performance across the assays was decreased for both single task and multi-task imputation approaches on the data with increased sparsity, however, the multi-task approaches remain clearly superior to XGB. Macau seems more robust towards increased sparsity compared to XGB-FN, as the decrease in MCC scores is less pronounced for this technique. Decreases in performance were more pronounced for assays where training labels were removed (the blue dots in 11B and C), whereas there was less impact on the scores for assays with fewer labels in the original dataset. This may be due to the overall structure of the ToxCast dataset whereby some compounds were tested in the majority of assays [21]. This means that assays with only a few labels are likely to contain mostly compounds that were tested in a large number of assays and therefore test compounds for those assays will have many experimental labels to use. Even if the overall sparsity is increased, the test compounds of these assays will still have a high number of experimental labels, so that little or no decrease in performance was observed. In particular, it may be that those auxiliary assays most closely related to the target assays (with fewer than 1000 labels) also had fewer than 1000 labels and hence the most important source of information would not have been removed. For a single assay, overall sparsity of the dataset may not be the main determinant for the success of multi-task imputation approaches and it could be that having information from at least some related assays may be crucial instead. This is explored further below.

### Role of assay relatedness on imputation models

**Fig. 12:**
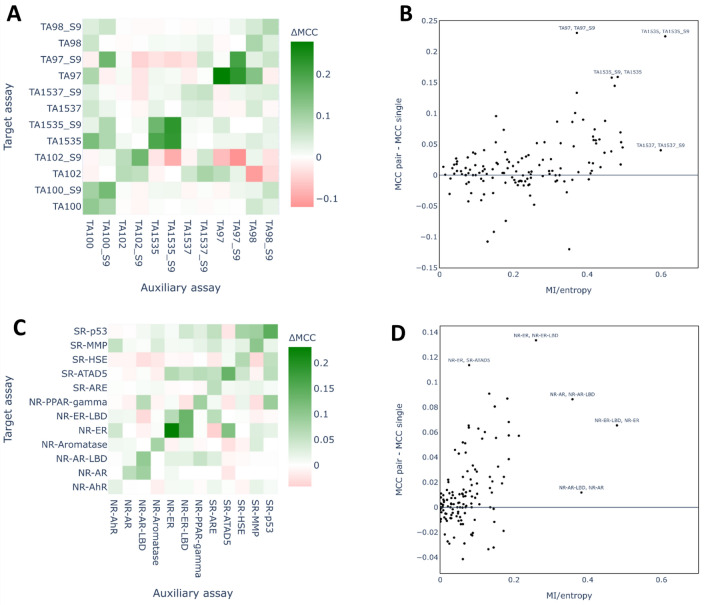
Results of the pairwise XGB-FN models and the assay relatedness analysis. **A**, **C** pairwise FN models for the Ames dataset (**A**) and Tox21 dataset (**C**). For each assay, pairwise FN models were trained with each of the remaining assays and, for each pair, 20 independent runs of the model were conducted using different random seeds and evaluated on the test set. To obtain the heatmap, the median MCC score was computed for each pair and the median MCC of the single task model for respective target assay was subtracted. The diagonals represent the differences in MCC score between the full FN model and the single task XGB model as a reference. **B**, **D** For each target assay-auxiliary assay pair the difference between MCC of pairwise FN model and single task model are plotted against the MI-entropy ratio of the two assays for the Ames dataset (**B**) and the Tox21 dataset (**D**).

The contributions of single assays to the overall success of the multi-task imputation were determined using pairwise FN models which were trained for each target assay with each of the remaining assays as auxiliary, in turn. Figure 12 reports the performance of the pairwise XGB-FN models compared to the single task models as difference heatmaps. The relationship between the difference in performance between pairwise XGB-FN and single task models and the relatedness of the assays, measured as the MI-entropy ratio, is also shown. The MI-entropy ratios for all assay pairs of the small datasets are given in Additional file 1: Fig. S4 as a heatmap.

For the Ames dataset, the pairwise XGB-FN models achieved a higher MCC score than the single task XGB models in many cases (as shown by the green cells in the heatmap outside the diagonal, Fig. 12A). The diagonal of the heatmap shows the difference between the MCC scores for the full FN models compared to single task models. Generally, the pairwise models did not achieve as high scores as the full FN models, with the exception of TA1535 with TA1535_S9. However, in a few cases, the pairwise FN model approximated the performance of the full FN model quite well (e.g. the MCC of the TA97 full model was 0.279 higher than the MCC of the single task model, whereas, the improvement of the pairwise model comprising TA97 and TA97_S9 was 0.230). In many cases, the pairwise FN model provided a substantial benefit (improvement over 0.05) compared to the single task model, even if this was smaller than that achieved for the full FN model. There were also many cases where the pairwise FN model showed very small differences compared to the single task models (shown by the white and very pale cells in the heatmap). Red cells indicate reduced performance of the pairwise models compared to the single task models. These cases were fairly rare overall, but occurred frequently in the assays with the fewest data points which also showed a high variance between different runs of a model (TA102, TA102_S9, TA97, TA97_S9). Unsurprisingly, the Ames strain results with and without S9 are highly correlated (for details see Additional file 1: Fig. S4) and this is reflected in the consistent increase in performance compared to the single task models for the pairs of the same bacteria strain, represented by the accumulation of green cells adjacent to the diagonal. Another key finding is that the four assays with the most data points (TA100, TA100_S9, TA98, TA98_S9) as auxiliary assays resulted in at least a moderate increase in performance in most cases, suggesting that the number of available experimentally determined data points impacts on the performance of the pairwise FN models.

The performances of the pairwise XGB-FN models on the Tox21 dataset are shown in Fig. 12C. Similar to the Ames dataset, the pairwise XGB-FN models achieved a higher MCC score than the single task XGB models in many cases. In two of the cases (NR-AR with NR-AR-LBD and with NR-PPAR-gamma) the pairwise model achieved a higher score than the full FN model. However, for most of the target assays the full FN model clearly performed better than any pairwise FN model. The Tox21 dataset contains two pairs of assays that measure the same target in a different test system (NR-AR/NR-AR-LBD and NR-ER/NR-ER-LBD) and it was therefore expected that these pairs would yield the best performing pairwise FN models. For NR-ER and NR-ER-LBD this was indeed the case, although other auxiliary assays yielded models of comparable performance (SR-ATAD5 for NR-ER and SR-ARE for NR-ER-LBD). NR-AR-LBD was the best auxiliary assay for NR-AR, but the same was not true for the opposite case (NR-PPAR-gamma was the best auxiliary assay for NR-AR-LBD). Some of the pairwise models performed worse than the respective single task models (red cells in Fig. 12B). For the target assays NR-PPAR-gamma and SR-HSE this occurred for many of the pairs, yet the full FN model performed better than the single task models, which can be attributed to the few auxiliary assays that resulted in improved models (SR-p53 and NR-AR-LBD for NR-PPAR-gamma, and SR-p53 for SR-HSE).

The pairwise FN model results showed that a single assay could have a large influence on the success of FN models, however, different auxiliary assays had very different effects. Figure 12B and D plot the relationship between a target assay and an auxiliary assay measured using the MI-entropy ratio, against the change in performance of the pairwise FN models over single task models for the Ames and Tox21 dataset, respectively.

For the Ames dataset, the improvements of pairwise FN models over single task models were not strongly correlated to the metric for assay relationships (Pearson correlation coefficient: 0.48) (Fig. 12B). Nonetheless, strong increases for pairwise models (improvements in MCC of over 0.1) only occurred for pairs where the MI-entropy ratio is above 0.3. Hence, it seems that a close relatedness between the target assay and the auxiliary assay was necessary but not sufficient for a strong effect of that auxiliary assay on the FN model. The pair TA1537 with TA1537_S9 represents a case where a strong relatedness of the assays resulted in an apparently small effect, however, the performance of single task XGB on TA1537 was already very high (median MCC: 0.691, Fig. 5A) and a larger MCC score on this dataset may be limited by the uncertainty in the toxicity labels.

Similar to the Ames dataset, an increase in the MI-entropy ratio tended to correspond with improvements of pairwise FN models over single task models on the Tox21 dataset, but the correlation was not very strong (Pearson correlation coefficient: 0.50). A striking exception from these trends was presented by the pair NR-AR-LBD with NR-AR where the MI-entropy ratio was very large (almost 0.4) but the change in MCC was very small (less than 0.02). However, the other pairs with high MI-entropy ratios (e.g. NR-ER with NR-ER-LBD) were amongst the pairs where the auxiliary assay had the strongest effects on the FN model. Overall, the values for the MI-entropy ratio were lower on the Tox21 dataset than for the Ames dataset, which could explain why the imputation models provided a larger numerical benefit on performance for the Ames dataset. The findings on both datasets suggest that the MI-entropy ratio might be a useful metric to estimate which auxiliary assays could provide the strongest benefit in a FN model. However, clearly a high value does not guarantee a strong benefit.

**Fig. 13:**
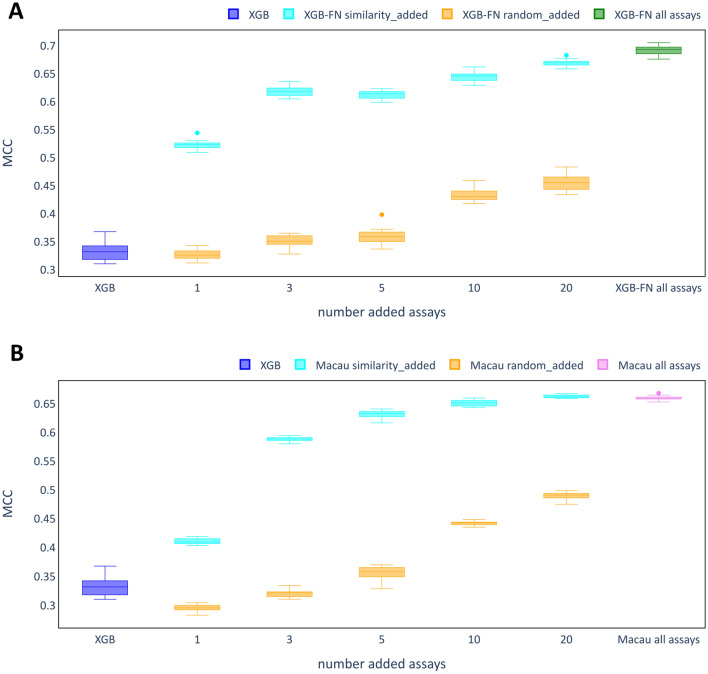
Auxiliary assay selection for the assay ‘TOX21-Aromatase-Inhibition’. Auxiliary assays were selected according to the MI-entropy ratio (‘similarity_added’) or randomly (‘random_added’). As a comparison, performance of XGB as a single task model and the respective multi-task model using all remaining auxiliary assays were included. Plotted are the MCC scores for the test set across 20 runs with different random seeds. **A** XGB-FN models. **B** Macau models

For the larger ToxCast dataset, a detailed analysis of a single target assay, ‘TOX21-Aromatase-Inhibition’, was conducted. Auxiliary assays were selected either randomly or using the MI-entropy ratio and the performance of the resulting models was compared. MCC score ranges of models are shown for XGB-FN models and Macau models in Fig. 13A and C, respectively.

For both XGB-FN and Macau, those models trained with auxiliary assays selected with the MI-entropy ratio criterion clearly outperformed those trained with randomly selected auxiliary assays. Macau models trained with the 20 most similar assays even performed slightly better than the models with all assays. For both XGB-FN and Macau, adding just one auxiliary assay provides a clear improvement over XGB models with a further strong increase after two more assays (three in total) were added. For the random assay selection, clear improvements were only observed after at least 10 assays were added.

Additional file 1: Table S7 provides an overview for all selected auxiliary assays in this experiment. For assays selected using the MI-entropy ratio the values for the metric range between 0.369 and 0.256, while those for the randomly selected ones range between 0.261 (one of the Top-20 assays was randomly selected) and 0.003. Relatively high MI-entropy values for some of the randomly selected assays may explain why clear improvements occurred also under this assay selection scheme.

## Discussion

### Comparison of traditional single task and multi-task models

For both the small-scale datasets, no significant difference in performance was found between single task and multi-task methods in terms of MCC. On the Tox21 dataset, both multi-task DNN and XGB-FN slightly outperformed the best single task approach, XGB. However, the opposite was found for the Ames dataset. The small differences in performance between the methods suggest that neither single task nor multi-task models are generally superior on these datasets.

An insightful feature of this study is the analysis of model performance across different runs of a model with different random initializations, but otherwise identical hyperparameters. Our results show that stochastic effects can have a large influence on the performance of machine learning models. For some of the assays and methods the variance in performance was larger than for others. Assays with relatively few data points or strongly imbalanced assays were found to have comparatively high variances. This can be explained by the fact that seemingly small variation in predictions made by a model can have a quite large impact on the MCC scores. Regarding different algorithms, DNNs (both single task and multi-task) were found to have relatively large variance across different assays. In our experience, limiting the comparison of different machine learning methods to a single score could lead to the occurrence of seemingly large differences in performance between models which are the result of pure chance, rather than meaningful differences. The repeated runs with different random seeds demonstrate that the observed differences between the single task and multi-task models are not significant when considering the variances of the models’ performances on the single assays.

FNs based on XGB models achieved the highest scores on the Tox21 dataset. However, the FN models based on RF and DNN performed slightly worse than their single task counterparts (see scores in the code repository). An explanation for this could be the poor performance of the single task models which can lead to inaccurate predicted labels in the first step of the FN approach which are then propagated into inaccurate predictions for the target assays. A clear improvement in performance was observed when experimentally determined toxicity labels for test compounds were used as inputs to the FN models. Experimentally determined labels indicate the true label of a compound with higher confidence than predictions, which may explain the surge in performance. These types of models are comparable to multi-task imputation models which are discussed further below, as they also use experimental information about test compounds.

To some extent, our findings seem to contradict reports on the superiority of multi-task models over single task models in the literature. The Merck Kaggle Challenge was won by an approach largely based on multi-task DNNs [2] with the results being further investigated by Xu et al. [38]. However, a direct comparison with our results is not possible due to differences in the datasets and the nature of the learning tasks which are classification here and were regression in the study by Xu et al. Nevertheless, even though on average multi-task DNNs outperformed single task DNNs in that study, the differences between the two approaches were also small. Mayr et al. found multi-task DNNs to outperform several single task algorithms, based on average performance, on the Tox21 dataset [], yet the differences were small and comparable to our findings (Table 2). Moreover, the selection of the metric for model evaluation may influence the conclusions. When using ROC-AUC instead of MCC as metric, both multi-task DNN and Macau outperformed the single task approaches on both the Ames and Tox21 dataset (Additional file 1: Table S5). For FN models using solely predicted activities as features in the test set, we found no consistent improvements over single task models (for MCC and ROC-AUC as metric), which is in contrast to previous reports on FNs [7, 8] and on the related approach using predicted bioactivity profiles by Norinder et al. [9].

### Comparison of single task and multi-task imputation models

For both the Ames and the Tox21 datasets, the best single task imputation model was outperformed by multi-task imputation models across all the assays. Numerically, the increases in MCC scores over single task models were larger for the Ames dataset than for the Tox21 dataset. The best three multi-task methods (Macau, XGB-FN, and multi-task DNN) achieved virtually the same average MCC scores on the Ames dataset. RF-FN and DNN-FN performed somewhat worse but were still clearly better than the single task models. On the Tox21 dataset, XGB-FN was the best method followed by multi-task DNN and the other FN methods also performed better than the single task models. However, Macau achieved lower MCC scores than the single task models, which is discussed below.

On the ToxCast dataset, XGB-FN and Macau were found to clearly outperform XGB models, whereas the multi-task DNN achieved higher scores than XGB for some assays and lower scores for others. In particular, for many assays a MCC score of 0 was obtained which hinted at the decision threshold being inappropriate as the result of imbalanced training data. The GHOST methodology was therefore applied to the models in order to optimize the thresholds individually for each assay and method. The optimized multi-task DNN model outperformed the single task XGB, however, it did not perform as well as XGB-FN and Macau. Across the different techniques, the GHOST approach was successful in improving MCC scores for assays that performed very poorly (MCC in many cases 0) when the default threshold was inappropriate. However, for assays performing well with the default threshold a slight decrease in MCC score was found for some assays. We conclude that the GHOST approach may be helpful when applied to imputation models, yet it should not necessarily be applied to all the assays. Situations where the GHOST approach seems useful are when assays have a poor MCC score, and the ROC-AUC score indicates reasonably good performance in ranking toxic compounds higher than non-toxic ones.

An advantage of FN models is the simplicity of implementation since they are based on existing single task QSAR architectures applied to multi-target datasets. However, the computational cost of FN models may be a limitation for very large datasets (i.e., many assays) as two models have to be trained for each assay. The FN approach as used in this study is somewhat reminiscent of the pQSAR approach [10]. Both approaches consist of two training steps, with the first step filling gaps in the dataset using single task models. The second step of the pQSAR model uses only the assay labels (known or predicted) as features rather than combining them with chemical descriptors as was done here.

The multi-task DNN used as an imputation method (i.e., on the assay-based splits) yielded competitive MCC scores on both the Ames and Tox21 datasets. In contrast to a multi-task DNN in its conventional use, the network is trained on all the compounds in the dataset (provided there is at least one label for the compound in the training set). An advantage of this technique compared to FN is that only a single model needs to be trained for the whole dataset rather than two for each assay. However, while the multi-task DNNs achieved high scores, the variance in performance due to changes in the random seed was relatively large. This behavior is undesirable as a single run may lead to a poorly performing model. This could be addressed by using ensembles of multiple runs, although this would increase the computational cost of the method. On the ToxCast dataset, the multi-task DNN achieved lower scores than FN and Macau models, even after applying GHOST. It could be that a more careful selection of hyperparameters including architecture may be required for better scores. In general, it may be challenging for a multi-task DNN to achieve competitive scores on a large number of assays with widely different numbers of training instances.

The Macau method achieved the highest average MCC score on the Ames dataset, but a poor average MCC score on the Tox21 dataset. Additional file 1: Figure S1 and Table S6 report ROC-AUC scores for the different imputation models where it can be seen that Macau performed best on both the Ames and Tox21 dataset using this metric. This suggests that an inappropriate decision threshold caused the low MCC scores for Macau on the Tox21 dataset, which might be remedied by using the GHOST approach. Macau was also found to perform well on the larger ToxCast dataset. An advantage of Macau compared to FN models is that less training time is required, as only a single model needs to be trained.

The findings of this study agree with previous studies reporting substantial benefits in performance of imputation models over single task models for regression, namely Alchemite and pQSAR [10, 11, 42]. Intriguingly, Alchemite and pQSAR are based on two conceptually different imputation techniques, and achieved approximately the same average score on the Novartis benchmark set [42]. Here, we used FN models as another imputation method and demonstrated its utility for classification tasks, both for datasets of small scale (Ames and Tox21 each having 12 assays) and a dataset of medium-large scale (ToxCast with 416 assays). The suitability of FN models for datasets of even larger dimensions (thousands of assays and millions of compounds) would need to be tested.

Having demonstrated the superior performance of multi-task techniques for imputation, investigations were then carried out that aimed to characterize the factors that contributed to the improvements. The investigations explored the effects of chemical similarity, the amount of data labels available, and the relatedness of the assays included in the imputation models. Similar detailed investigations have not been reported in the literature to our knowledge.

The multi-task models achieved higher scores than single task models for test compounds in all similarity bins (representing different degrees of chemical similarity to training compounds). The largest increase was observed for compounds with low chemical similarity to the training compounds, on which the single task models performed poorly. This effect was stronger for the Ames dataset, but was also observed for the Tox21 dataset for assays where the single task QSAR model performed particularly poorly on chemically dissimilar compounds. We therefore conclude that not only do multi-task imputation models perform better than single task imputation models, but by leveraging known auxiliary assay data even in less represented chemical space they may also possess a wider applicability domain and increase the chemical space for which a model makes reliable predictions.

Perhaps unsurprisingly, our results showed that multi-task models outperformed single task models by a wider margin for compounds with many toxicity labels. Nevertheless, both multi-task DNN and Macau outperformed XGB models on the Ames dataset for compounds with no or just one toxicity label present in the training set, indicating that very little information may be sufficient to observe improvement over single task QSAR models. On the ToxCast dataset it was confirmed that increasing sparsity (by removing labels from the training set) decreases performance of multi-task imputation models, yet the same is true for single task models and hence the multi-task techniques still provided a clear benefit over single task models. The experiments on pairwise FN models demonstrated that knowing the label of just a second toxicity assay can improve the predictions substantially, although this depends on which assay is used as auxiliary assay. Using the assay ‘TOX21-Aromatase-Inhibition’ from the ToxCast dataset, it was demonstrated that the MI-entropy ratio can be used to successfully identify auxiliary assays yielding large benefits for a given target assay. Clearly, both the type and amount of information in the form of experimental labels for auxiliary assays determine the success of imputation models. Having many auxiliary labels and, in particular, labels of closely related assays leads to the largest improvements in model performance. The MI-entropy ratio introduced in this work provides a useful means to formalize and quantify the concept of relatedness between different toxicity assays.

## Conclusions

This study found little difference in performance between single task and multi-task QSAR models in classical QSAR settings using traditional compound-based splits. However, multi-task models clearly outperformed the single task models when imputation was used, as demonstrated using the assay-based splits, so that the inclusion of data from other toxicity endpoints for test compounds may yield superior QSAR models. The study also suggests that multi-task imputation models may be able to make reliable predictions for a wider chemical space when compared to traditional QSAR models. Hence, multi-task imputation models may be particularly useful in situations where the use of traditional QSAR models is limited due to test compounds falling outside of the applicability domain of a single model. However, it is important to acknowledge that imputation is fundamentally different from the more conventional approach since it uses experimentally determined information about toxicity endpoints for the test compounds. This is likely the reason why these models can achieve much better performance scores.

Naturally, imputation approaches are restricted to predict toxicity for compounds where toxicity has already been measured for other endpoints. The FN models that used predicted activities as features for the test compounds were not found to outperform single task models on the datasets studied here, therefore, when no experimental data about test compounds is available multi-task DNNs may be the best choice. Depending on the dataset, these models might provide a slight benefit in performance over single task models. Furthermore, the use of a multi-task DNN can save computation time compared to the training of single task QSAR models for each individual endpoint.

Although our datasets have focused on toxicity endpoints, we believe the results are applicable to other biological endpoints of interest. In conclusion, multi-task imputation models have the potential to improve the performance of QSAR models used in practice and to extend their domain of applicability to make predictions for dissimilar molecules.

## Supplementary Information

**Additional file 1: Tables S1–S4.** The hyperparameter values tested on the grid search for each machine learning algorithm. **Table S5.** ROC-AUC scores for single task and multi-task models using compound-based splits. **Table S6, Figures. S1, S2.** ROC-AUC scores for the imputation models using assay-based splits. **Figure S3.** Assay-wise comparisons of MCC scores with and without using the GHOST approach. **Figure S4.** Heatmaps showing the relatedness measured between the assays as ratio of mutual information and entropy of the target assay. **Table S7.** Auxiliary assays selected for ‘TOX21-Aromatase-Inhibition’.

## Data Availability

The datasets and code supporting the conclusions of this article are available in the Github repository (https://github.com/mowal/Imputation_Paper). The repository contains the original data, the dataset splits used, the code to run the algorithms and the results for each model trained with MCC, F1 and ROC-AUC values.

## Abbreviations

ANN: Artificial neural network
DNN: Deep neural network
EPA: U.S. Environmental Protection Agency
FN: Feature Net
GHOST: Generalized threshold shifting procedure
ISSSTY: Istituto Superiore di Sanità Salmonella Typhymurium
MCC: Matthews correlation coefficient
MI: Mutual information
RF: Random forest
XGB: XGBoost

## References

[1] Caruana R (1997). Multitask learning. Mach Learn.

[2] Mayr A, Klambauer G, Unterthiner T, Hochreiter S (2016). DeepTox: toxicity prediction using deep learning. Front Environ Sci.

[3] Ma J, Sheridan RP, Liaw A (2015). Deep neural nets as a method for quantitative structure-activity relationships. J Chem Inf Model.

[4] Simm J, Arany A, Zakeri P, et al (2015) Macau: scalable Bayesian multi-relational factorization with side information using MCMC. arxiv:150904610v2

[5] de la Vega de León A, Chen B, Gillet VJ (2018). Effect of missing data on multitask prediction methods. J Cheminform.

[6] Trapotsi MA, Mervin LH, Afzal AM (2021). Comparison of chemical structure and cell morphology information for multitask bioactivity predictions. J Chem Inf Model.

[7] Varnek A, Gaudin C, Marcou G (2009). Inductive transfer of knowledge: application of multi-task learning and Feature Net approaches to model tissue-air partition coefficients. J Chem Inf Model.

[8] Sosnin S, Karlov D, Tetko IV, Fedorov MV (2019). Comparative study of multitask toxicity modeling on a broad chemical space. J Chem Inf Model.

[9] Norinder U, Spjuth O, Svensson F (2020). Using predicted bioactivity profiles to improve predictive modeling. J Chem Inf Model.

[10] Martin EJ, Polyakov VR, Zhu XW (2019). All-Assay-Max2 pQSAR: activity predictions as accurate as four-concentration IC50s for 8558 novartis assays. J Chem Inf Model.

[11] Whitehead TM, Irwin BWJ, Hunt P (2019). Imputation of assay bioactivity data using deep learning. J Chem Inf Model.

[12] Irwin BWJ, Levell JR, Whitehead TM (2020). Practical applications of deep learning to impute heterogeneous drug discovery data. J Chem Inf Model.

[13] Martin EJ, Polyakov VR, Tian L, Perez RC (2017). Profile-QSAR 2.0: kinase virtual screening accuracy comparable to four-concentration IC50s for realistically novel compounds. J Chem Inf Model.

[14] Cherkasov A, Muratov EN, Fourches D (2014). QSAR modeling: Where have you been? Where are you going to?. J Med Chem.

[15] ISSSTY database. https://www.iss.it/isstox. Accessed 25 May 2021

[16] OECD (1997). Test No. 471: bacterial reverse mutation test. J Phys A Math Gen.

[17] Benigni R, Battistelli CL, Bossa C (2013). New perspectives in toxicological information management, and the role of ISSTOX databases in assessing chemical mutagenicity and carcinogenicity. Mutagenesis.

[18] Mortelmans K, Zeiger E (2000). The Ames Salmonella/microsome mutagenicity assay. Mutat Res Mol Mech Mutagen.

[19] Tox21 Challenge dataset. https://tripod.nih.gov/tox21/challenge/data.jsp. Accessed 25 May 2021

[20] Huang R, Xia M, Sakamuru S (2016). Modelling the Tox21 10 K chemical profiles for in vivo toxicity prediction and mechanism characterization. Nat Commun.

[21] Richard AM, Judson RS, Houck KA (2016). ToxCast chemical landscape: paving the road to 21st century toxicology. Chem Res Toxicol.

[22] Wu Z, Ramsundar B, Feinberg EN (2018). MoleculeNet: a benchmark for molecular machine learning. Chem Sci.

[23] RDKit: Open-source cheminformatics. http://www.rdkit.org. Accessed 25 May 2021

[24] Swain M MolVS. https://github.com/mcs07/MolVS. Accessed 25 May 2021

[25] Weininger D (1988). SMILES, a chemical language and information system: 1: introduction to methodology and encoding rules. J Chem Inf Comput Sci.

[26] Heller SR, McNaught A, Pletnev I (2015). InChI, the IUPAC international chemical identifier. J Cheminform.

[27] Rogers D, Hahn M (2010). Extended-connectivity fingerprints. J Chem Inf Model.

[28] Svetnik V, Liaw A, Tong C (2003). Random forest: a classification and regression tool for compound classification and QSAR modeling. J Chem Inf Comput Sci.

[29] Python Software Foundation Python Language Reference, version 3. https://www.python.org/. Accessed 25 May 2021

[30] Pedregosa F, Varoquaux G, Gramfort A (2011). Scikit-learn: machine learning in python. J Mach Learn Res.

[31] Meir R, Rätsch G (2003) An introduction to boosting and leveraging. Lect Notes Comput Sci. 10.1007/3-540-36434-x_4

[32] Friedman JH (2001). Greedy function approximation: a gradient boosting machine. Ann Stat.

[33] Chen T, Guestrin C (2016) XGBoost: a scalable tree boosting system. In: Proceedings of the ACM SIGKDD international conference on knowledge discovery and data mining. pp 785–794

[34] LeCun Y, Bengio Y, Hinton G (2015). Deep learning. Nature.

[35] Abadi M, Barham P, Chen J, et al (2016) TensorFlow: A System for Large-scale Machine Learning. In: Proceedings of the 12th USENIX Conference on operating systems design and implementation. USENIX Association, Berkeley, CA, USA, pp 265–283

[36] Chollet F, others (2015) Keras. https://keras.io. Accessed 25 May 2021

[37] Davis IL, Stentz A (1995) Sensor fusion for autonomous outdoor navigation using neural networks. In: Proceedings 1995 IEEE/RSJ international conference on intelligent robots and systems. Human robot interaction and cooperative robots. IEEE Computer Society Press, pp 338–343

[38] Xu Y, Ma J, Liaw A (2017). Demystifying multitask deep neural networks for quantitative structure−activity relationships. J Chem Inf Model.

[39] Esposito C, Landrum GA, Schneider N (2021). GHOST: adjusting the decision threshold to handle imbalanced data in machine learning. J Chem Inf Model.

[40] Song B, Zhang G, Zhu W, Liang Z (2014). ROC operating point selection for classification of imbalanced data with application to computer-aided polyp detection in CT colonography. Int J Comput Assist Radiol Surg.

[41] Shannon CE (1948). A Mathematical theory of communication. Bell Syst Tech J.

[42] Irwin BWJ, Mahmoud S, Whitehead TM et al (2020) Imputation versus prediction: applications in machine learning for drug discovery. Futur Drug Discov 2:FDD38. 10.4155/fdd-2020-0008

