# Supplementary material for: Analysis of the benefits of imputation models over traditional QSAR models for toxicity prediction
Source: J Cheminform. 2022 Jun 7;14:32. doi: 10.1186/s13321-022-00611-w (PMC9172131; doi:10.1186/s13321-022-00611-w)
Supplement: Supplementary file 1 — Additional file 1: Tables S1–S4. The hyperparameter values tested on the grid search for each machine learning algorithm. Table S5. ROC-AUC scores for single task and multi-task models using compound-based splits. Table S6, Figures. S1, S2. ROC-AUC scores for the imputation models using assay-based splits. Figure S3. Assay-wise comparisons of MCC scores with and without using the GHOST approach. Figure S4. Heatmaps showing the relatedness measured between the assays as ratio of mutual information and entropy of the target assay. Table S7. Auxiliary assays selected for ‘TOX21-Aromatase-Inhibition’. [file 13321_2022_611_MOESM1_ESM.docx]

Analysis of the benefits of imputation models over traditional QSAR models for toxicity prediction

Moritz Walter^1^, Luke N Allen^1^, Antonio de la Vega de León^1^, Samuel J Webb^2^, Valerie J Gillet^1^

1 Information School, University of Sheffield, 211 Portobello, Sheffield S1 4DP, United Kingdom

2 Lhasa Limited, Granary Wharf House, 2 Canal Wharf, Leeds LS11 5PS, United Kingdom

**Table of content**

Table S1. Random Forest hyperparameters considered in optimization.

Table S2. XGBoost hyperparameters considered in optimization.

Table S3. Deep Neural Network hyperparameters considered in optimization.

Table S4. Macau hyperparameters considered in optimization.

Table S5 Median ROC-AUC values and interquartile ranges for compound-based splits

Table S6. Median ROC-AUC values and interquartile ranges for assay-based splits.

Table S7. Selected auxiliary assays for TOX21-Aromatase-Inhibition

Figure S1. Performance of imputation models on the small datasets evaluated using ROC-AUC.

Figure S2. Performance of imputation models on the ToxCast dataset using ROC-AUC.

Figure S3. Performance of the imputation models using the GHOST approach

Figure S4. Ratio of Mutual Information (MI) and entropy of the target assay for Ames and Tox21 assays.

The tables S1-S4 provide the hyperparameters considered for optimization in a grid search. The optimal values found for each combination of split type, algorithm and assay can be found in the code repository (https://github.com/mowal/Imputation_Paper).

**Table S1. Random Forest hyperparameters considered in optimization**.

For each hyperparameter modified in the grid search of Random Forest models, a brief explanation is provided alongside the values tested during the grid search.

| **Hyperparameter** | **Explanation** | **Grid search values** |
| --- | --- | --- |
| n_estimators | Number of trees in the Random Forest model | 100, 500 |
| class_weight | Assigns weights to instances of different classes | None, balanced, balanced_subsample |
| max_features | Number of randomly selected features that are considered to find the best split | sqrt, 0.25 |

**Table S2. XGBoost hyperparameters considered in optimization**.

For each hyperparameter modified in the grid search of XGBoost models, a brief explanation is provided alongside the values tested during the grid search.

| **Hyperparameter** | **Explanation** | **Grid search values** |
| --- | --- | --- |
| Num_round | Number of sequential trees that are trained | 300, 700 |
| eta | Learning rate that shrinks the size of the update of predictions after each boosting step | 0.1, 0.3, 0.5 |
| Colsample_bytree | Proportion of features used within each individual tree | 0.5, 0.7 |
| Alpha | Strength of L1 regularization of the weights per leaf | 0, 1 |
| lambda | Strength of L2 regularization of the weights per leaf | 1, 10 |
| Scale_pos_weight | Assign weights to instances of different classes | 1, weighted |

**Table S3. Deep Neural Network hyperparameters considered in optimization**.

For each hyperparameter modified in the grid search of Deep Neural Network models, a brief explanation is provided alongside the values tested during the grid search. ST: single task, MT: multi-task

| **Hyperparameter** | **Explanation** | **Grid search values** |
| --- | --- | --- |
| Hidden layers | Number of hidden layers in the network | 1, 2, 3 |
| Nodes per hidden layer | Number of nodes in each hidden layer | 1024, 2048 |
| Learning rate | Size of the updates to the network parameters per training step | 0.0003, 0.001, 0.003 |
| Dropout | Proportion of neurons randomly removed in each training step | 0, 0.2 |
| L2 regularization | Strength of L2 regularization on the weights per node | 0.0001, 0.001 |
| Batch size | Size of batches used for each update of weights with the Adam optimizer | 10, 50 |
| Number of epochs | Number of epochs (each data point is used once for training per epoch) | 3, 5, 10 |
| Class weight | Assigns weights to instances of different classes | 1, weighted [ST]  1, 3, 5, 15 [MT] |

**Table S4. Macau hyperparameters considered in optimization**

For each hyperparameter modified in the grid search of Macau models, a brief explanation is provided alongside the values tested during the grid search.

| **Hyperparameter** | **Explanation** | **Grid search values** |
| --- | --- | --- |
| Num_latent | Number of dimensions used for the latent space | 16, 32, 64 |
| nsamples | Number of samples drawn from the Gibbs sampler | 800, 1600, 3200 |
| burnin | Number of burn-in samples from the Gibbs sampler that are discarded | 200, 400, 800 |

**Table S5. Median ROC-AUC values and interquartile ranges for compound-based splits**. Median scores and interquartile-ranges for each technique and dataset on the test set across the 20 random seeds. Before computing the median, the mean across the different assays for a single run was calculated. The best model for each dataset in bold.

|  |  | Ames | Tox21 |
| --- | --- | --- | --- |
| Single task | RF | 0.866 (0.865-0.866) | 0.818 (0.817-0.820) |
|  | XGB | 0.863 (0.862-0.864) | 0.811 (0.811-0.813) |
|  | ST-DNN | 0.842 (0.840-0.844) | 0.788 (0.784-0.790) |
| Multi-task | RF-FN | 0.862 (0.860-0.863) | 0.803 (0.802-0.804) |
|  | XGB-FN | 0.853 (0.852-0.855) | 0.812 (0.811-0.813) |
|  | ST-DNN-FN | 0.836 (0.835-0.839) | 0.774 (0.770-0.779) |
|  | MT-DNN | 0.871 (0.868-0.874) | 0.825 (0.822-0.830) |
|  | Macau | **0.873** (0.872-0.874) | **0.830** (0.830-0.831) |

**Table S6 Median ROC-AUC values and interquartile ranges for assay-based splits.** Single task models are included as a benchmark. Median scores and interquartile ranges for each technique and dataset on the test set across 20 different random seeds. Before computing the median, the mean across the different assays for a single run was calculated. The best model for each dataset is in bold.

|  |  | Ames | Tox21 |
| --- | --- | --- | --- |
| Single task | RF | 0.848 (0.846-0.849) | 0.817 (0.815-0.818) |
|  | XGB | 0.848 (0.846-0.848) | 0.805 (0.804-0.807) |
|  | ST-DNN | 0.831 (0.829-0.833) | 0.780 (0.778-0.783) |
| Multi-task | XGB-FN | 0.923 (0.923-0.924) | 0.866 (0.865-0.867) |
|  | MT-DNN | 0.935 (0.934-0.938) | 0.867 (0.864-0.870) |
|  | Macau | **0.944** (0.943-0.945) | **0.888** (0.887-0.888) |

**Table S7 Selected auxiliary assays for TOX21-Aromatase-Inhibition.** Assays were either selected according to their MI-entropy ratio or randomly.

| **Assays selected according to MI-entropy ratio** | **MI-entropy ratio** | **Randomly selected assays** | **MI-entropy ratio** |
| --- | --- | --- | --- |
| TOX21_TR_LUC_GH3_Antagonist | 0.369 | ATG_RARb_TRANS_dn | 0.018 |
| TOX21_AR_BLA_Antagonist_ratio | 0.308 | TOX21_MMP_ratio_up | 0.019 |
| BSK_SAg_CD40_down | 0.295 | ATG_Oct_MLP_CIS_up | 0.124 |
| BSK_SAg_CD69_down | 0.289 | ATG_DR4_LXR_CIS_dn | 0.136 |
| TOX21_AR_LUC_MDAKB2_Antagonist2 | 0.287 | ATG_HSE_CIS_dn | 0.003 |
| TOX21_AR_LUC_MDAKB2_Antagonist | 0.284 | OT_ER_ERaERa_1440 | 0.007 |
| BSK_LPS_SRB_down | 0.279 | ATG_Ets_CIS_dn | 0.048 |
| BSK_4H_Pselectin_down | 0.276 | BSK_3C_HLADR_down | 0.253 |
| BSK_3C_Proliferation_down | 0.270 | BSK_KF3CT_IP10_down | 0.209 |
| BSK_3C_Vis_down | 0.270 | BSK_hDFCGF_TIMP1_down | 0.206 |
| NCCT_HEK293T_CellTiterGLO | 0.269 | TOX21_HSE_BLA_agonist_ch2 | 0.043 |
| BSK_SAg_SRB_down | 0.267 | TOX21_VDR_BLA_agonist_ch2 | 0.004 |
| BSK_3C_SRB_down | 0.267 | NVS_ADME_hCYP19A1 | 0.073 |
| TOX21_FXR_BLA_antagonist_ratio | 0.267 | APR_HepG2_MicrotubuleCSK_24h_dn | 0.047 |
| BSK_CASM3C_Proliferation_down | 0.265 | BSK_SAg_MIG_down | 0.246 |
| BSK_SAg_CD38_dow | 0.261 | BSK_LPS_MCP1_down | 0.261 |
| BSK_LPS_MCP1_dow | 0.261 | ATG_NRF2_ARE_CIS_up | 0.121 |
| BSK_3C_IL8_down | 0.261 | BSK_3C_uPAR_down | 0.214 |
| BSK_SAg_IL8_down | 0.257 | NHEERL_ZF_144hpf_TERATOSCORE_up | 0.131 |
| BSK_SAg_MCP1_down | 0.256 | TOX21_PPARd_BLA_Antagonist_ch1 | 0.012 |


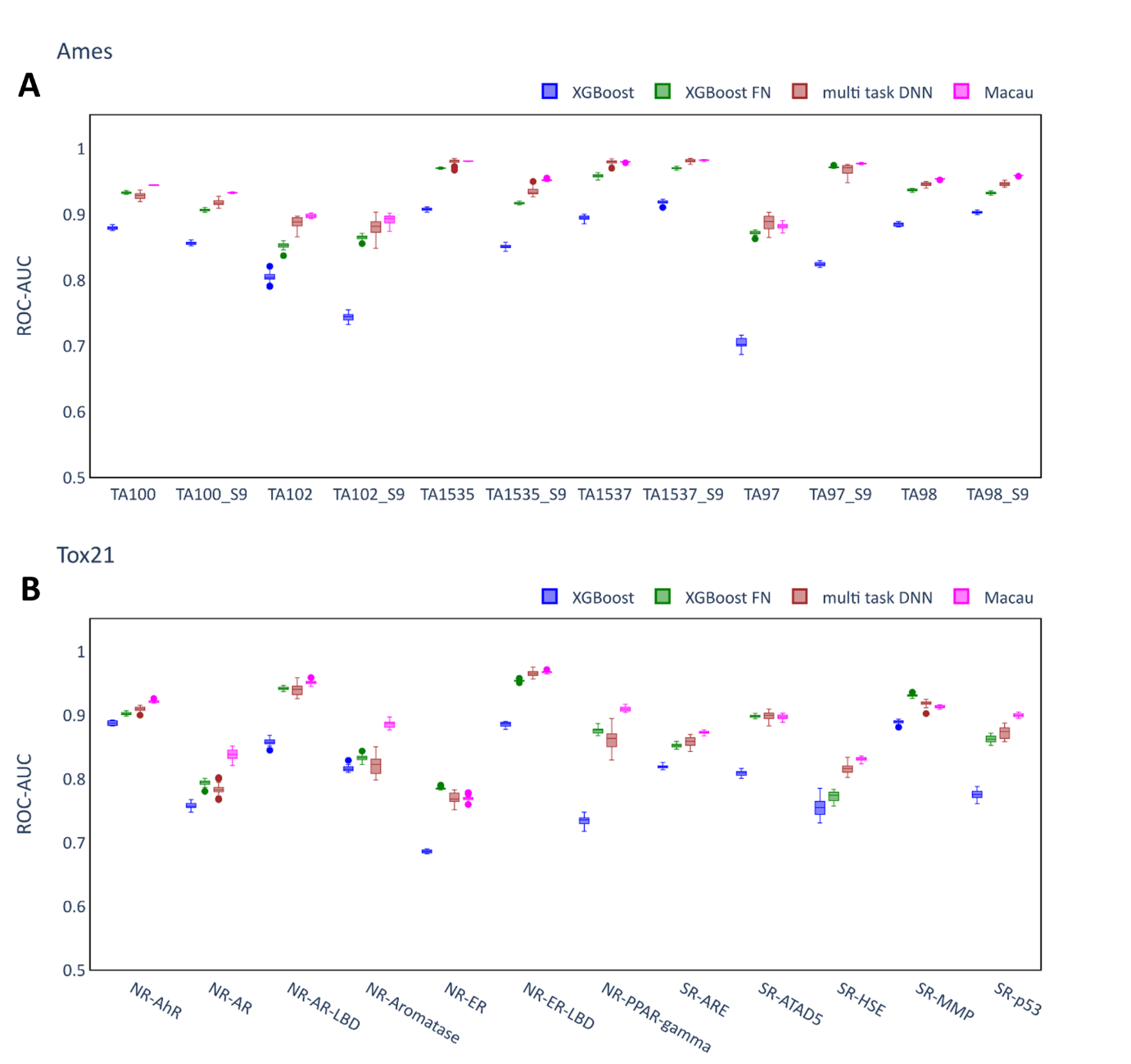


**Figure S1** **Performance of imputation models on the small datasets evaluated using ROC-AUC.** A: Ames dataset. B: Tox21 dataset. Each box summarizes the ROC-AUC scores of 20 independent runs of the model on the test set with identical hyperparameters but different random seeds. The XGB models (best performing single task QSAR model based on MCC) are included as a benchmark. Only the best performing Feature Net model (XGB-FN) is included in this plot


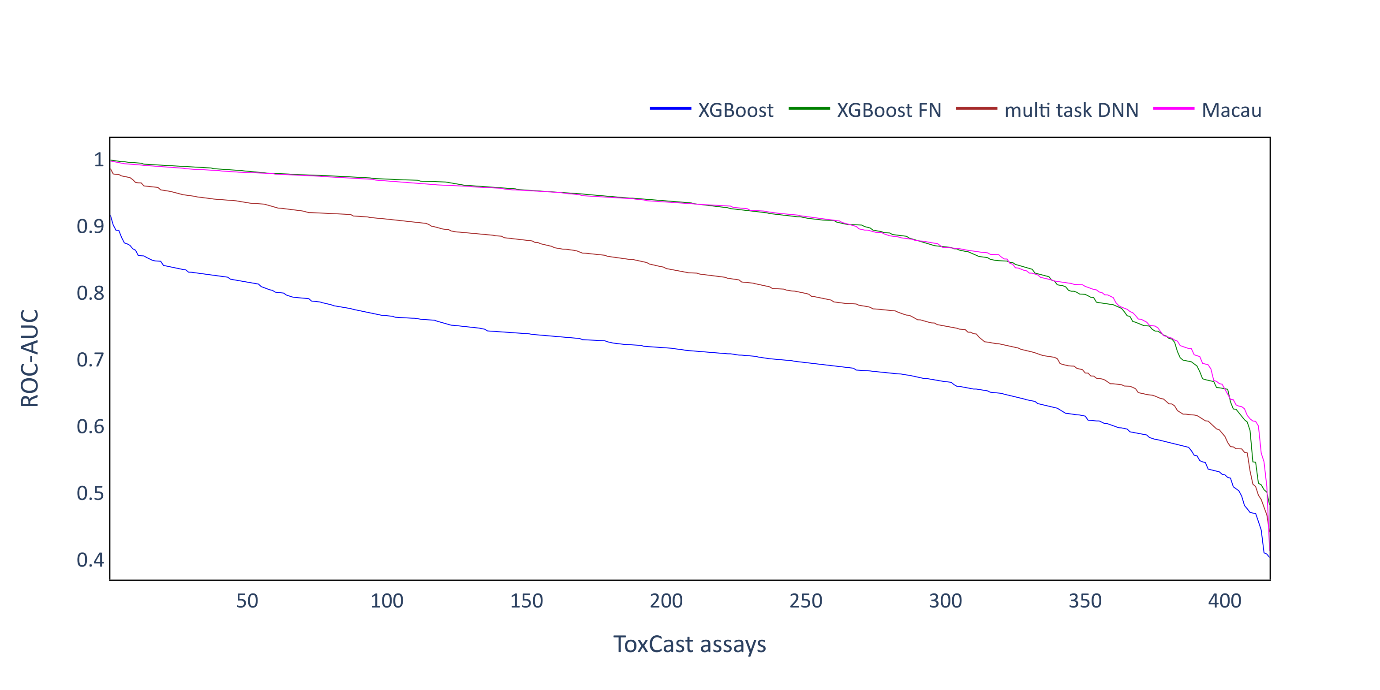


**Figure S2 Performance of imputation models on the ToxCast dataset using ROC-AUC.** ROC-AUC scores obtained for the individual assays are sorted in descending order and plotted as a line.


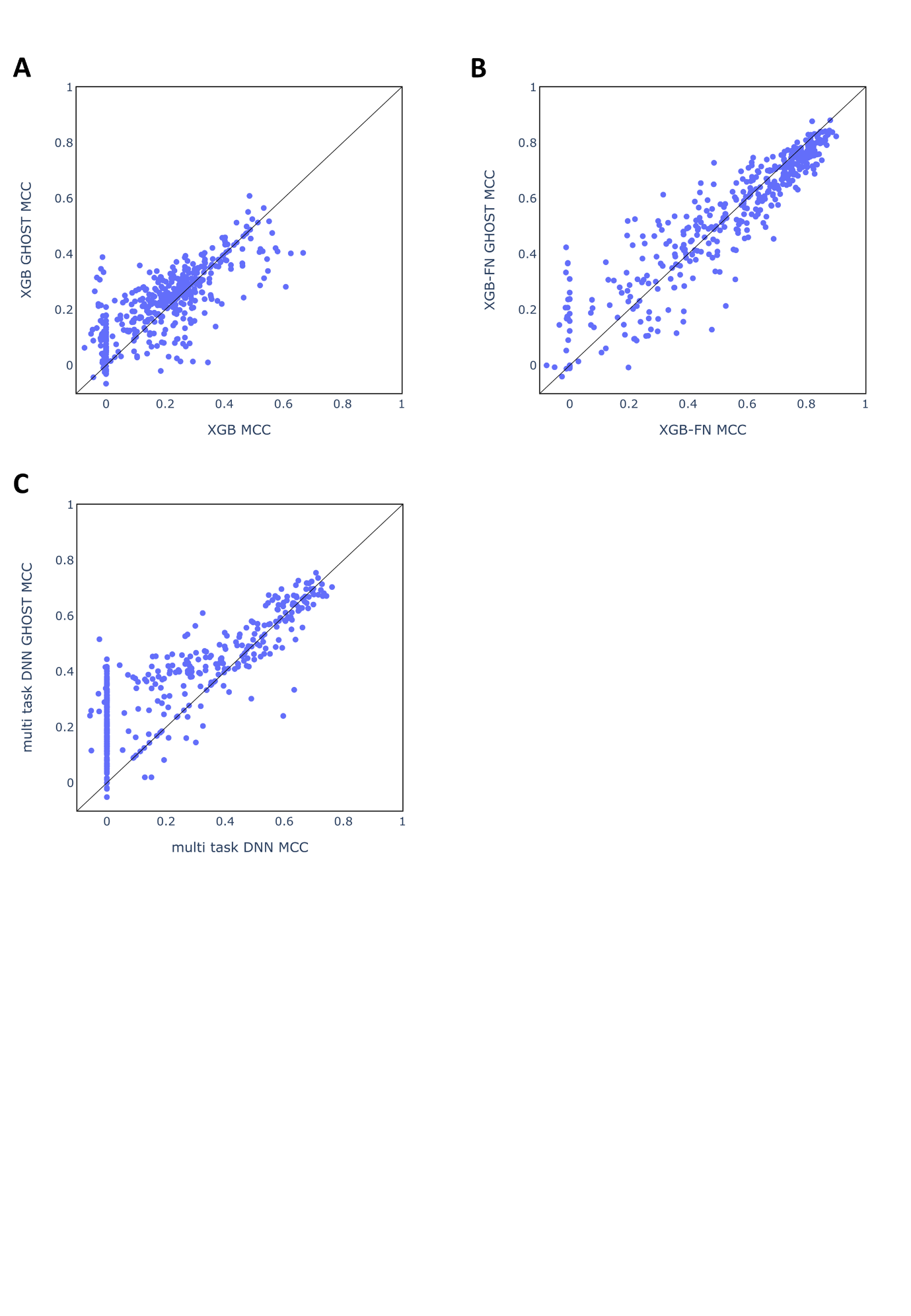


**Figure S3 Performance of the imputation models using the GHOST approach.** Scatter plots contrasting MCC scores with or without the GHOST approach for single assays of the ToxCast dataset. A: XGB, B: XGB-FN, C: multi-task-DNN. The scatter plot for Macau is depicted in Figure 7B.


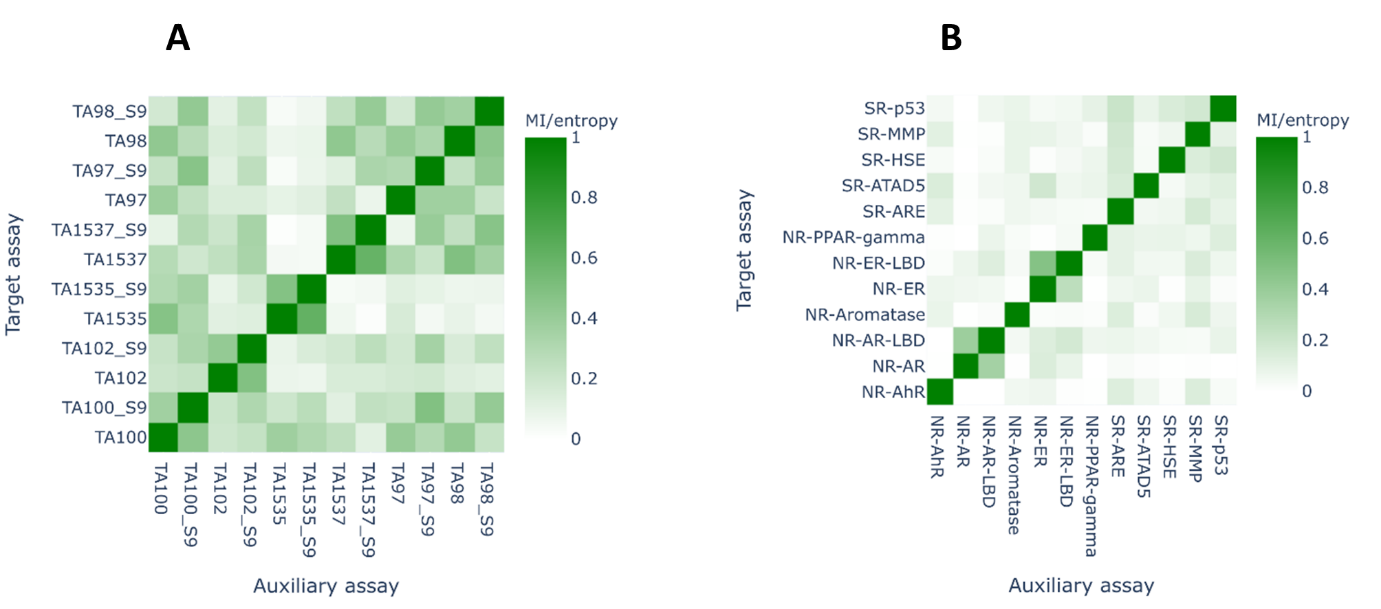


**Figure S4. Ratio of Mutual Information (MI) and entropy of the target assay for Ames (A) and Tox21 (B) assays.**
